# MTCH2 Deficiency Promotes E2F4/TFRC‐Mediated Ferroptosis and Sensitizes Colorectal Cancer Liver Metastasis to Sorafenib

**DOI:** 10.1002/advs.202500019

**Published:** 2025-07-02

**Authors:** Pu Xing, Jiangbo Chen, Hao Hao, Xiaowen Qiao, Xinying Yang, Kai Weng, Jie Chen, Lin Song, Tianqi Liu, Yifan Hou, Tongkun Song, Yumeng Ran, Bo Chen, Hong Yang, Wei Zhao, Zaozao Wang, Jiabo Di, Beihai Jiang, Xiangqian Su

**Affiliations:** ^1^ Key Laboratory of Carcinogenesis and Translational Research (Ministry of Education/Beijing) Department of Gastrointestinal Surgery IV Peking University Cancer Hospital & Institute Beijing 100142 China; ^2^ School of Basic Medical Sciences Peking University Health Science Center Beijing 100191 China; ^3^ Department of Pathology Peking Union Medical College Hospital Peking Union Medical College and Chinese Academy of Medical Science Beijing 100730 China; ^4^ Department of Gastrointestinal Surgery Peking University Cancer Hospital (Inner Mongolia Campus) & Affiliated Cancer Hospital of Inner Mongolia Medical University Hohhot 010010 China; ^5^ Ningxia Clinical Research Institute People's Hospital of Ningxia Hui Autonomous Region Ningxia Medical University Yinchuan China; ^6^ State Key Laboratory of Holistic Integrative Management of Gastrointestinal Cancers Department of Gastrointestinal Surgery IV Peking University Cancer Hospital & Institute Beijing 100142 China

**Keywords:** colorectal cancer, ferroptosis, liver metastasis, MTCH2, sorafenib

## Abstract

Ferroptosis is a specific type of lipid peroxide‐mediated cell death which is crucial in tumor suppression. While the mitochondrial carrier homolog 2 (MTCH2) is implicated in lipid homeostasis and mitochondrial metabolism, its role in ferroptosis and colorectal cancer (CRC) remains uncharacterized. Here, MTCH2 is identified as a crucial regulator of ferroptosis in CRC progression. Clinically, high expression of MTCH2 in CRC tissues predicts poor prognosis. Functionally, loss of MTCH2 inhibits azoxymethane (AOM)/dextran sodium sulfate (DSS)‐induced colorectal tumorigenesis in MTCH2^cKO^ mice and leads to accumulation of ferrous ion and enhances ferroptosis of CRC in vitro and in vivo. Mechanistically, MTCH2 deficiency promotes the proteasome‐dependent ubiquitination of E2F4 and attenuates transcriptional inhibition of transferrin receptor (TFRC) by E2F4, ultimately facilitating TFRC‐mediated ferroptosis in CRC cells. Moreover, MTCH2 depletion combined with sorafenib treatment synergistically triggers ferroptosis, suppresses liver metastasis, and effectively eradicates tumors in liver metastasis foci. Taken together, This study reveals the mechanism of MTCH2 deficiency‐induced ferroptosis to inhibit the progression of CRC and supports a potential therapeutic strategy targeting the MTCH2/E2F4/TFRC signaling axis in CRC patients with liver metastasis.

## Graphical abstract

This study identifies MTCH2 as a crucial regulator of ferroptosis in colorectal cancer (CRC) progression. High expression of MTCH2 is correlated with poor prognosis in CRC patients. Furthermore, MTCH2 depletion induces ferroptosis to suppress CRC liver metastasis via the E2F4/TFRC axis and sensitizes tumors to sorafenib treatment, supporting MTCH2 as a novel target to improve antitumor therapy.

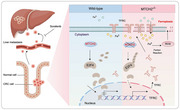

## 1 Introduction

Colorectal cancer (CRC) is the third most prevalent cancer and the second leading cause of cancer‐related mortality worldwide.^[^ ^1^ ^]^ As the primary cause of death in CRC,^[^ ^2^ ^]^ distant metastasis occurs in ≈50% of CRC patients, with liver metastasis being particularly common.^[^ ^3^ ^]^ However, the molecular mechanisms of CRC metastasis remain poorly understood and must be unraveled if we are to develop new interventional therapies to improve outcomes in patients.

Ferroptosis is a newly discovered form of programmed cell death marked by the iron‐mediated accumulation of lipid peroxidation products.^[^ ^4^ ^]^ Widespread attention has been paid to the significant therapeutic effects of ferroptosis in various conditions, including acute myocardial infarction, tumorigenesis, and neurodegenerative diseases.^[^ ^5^, ^6^, ^7^ ^]^ Therefore, potential ferroptosis‐related therapeutic strategies for multiple diseases are highly desired. Iron is involved in many cellular biological processes, including DNA repair, oxygen transport, and enzymatic reactions.^[^ ^8^, ^9^ ^]^ The disruption of normal iron transport may lead to the accumulation of intracellular iron, which drives the production of intracellular reactive oxygen species (ROS), triggers lipid peroxidation, and promotes the occurrence of ferroptosis.^[^ ^10^ ^]^ TFRC is essential for transporting iron into cells, and TFRC upregulation reportedly increases the sensitivity of tumor cells to ferroptosis by increasing intracellular iron ion levels.^[^ ^11^ ^]^ Increasing evidence has suggested that TFRC is a specific indicator of ferroptosis, modulating cellular iron uptake in cells.^[^ ^12^, ^13^ ^]^ The critical role of TFRC in ferroptosis suggests that inducing ferroptosis by upregulating TFRC levels could suppress tumor development.^[^ ^14^ ^]^ Therefore, identifying key molecules and mechanisms involved in TFRC regulation may support the development of new tumor‐treatment strategies.

The outer mitochondrial protein MTCH2 (SLC25A50) is a member of the solute carrier family and is critical for mitochondrial metabolism, apoptosis, and stem cell differentiation.^[^ ^15^, ^16^, ^17^ ^]^ Accumulating evidence has demonstrated that changes in MTCH2 levels can lead to various health conditions, including obesity, Alzheimer's disease, and embryological developmental anomalies.^[^ ^18^, ^19^, ^20^ ^]^ Guna et al. suggested that MTCH2 is a critical mitochondrial outer membrane insertase that regulates the levels of α‐helical transmembrane proteins, providing a potential mechanistic explanation for its involvement in diverse diseases.^[^ ^21^ ^]^ Furthermore, multiple studies have reported that MTCH2 is associated with the progression of various malignant tumors and might be a promising therapeutic target.^[^ ^22^ ^]^ However, the role and mechanism of MTCH2 in tumors are controversial, with several studies showing that it suppresses tumorigenesis by inducing apoptosis,^[^ ^23^, ^24^ ^]^ and others arguing that it promotes tumor progression by enhancing cellular metabolism.^[^ ^25^, ^26^ ^]^

In this study, we sought to elucidate the clinical significance of MTCH2 in CRC patients and investigate its role in ferroptosis. We found that high expression of MTCH2 was correlated with poor prognosis in CRC patients. Subsequently, the results of cytological experiments showed that knockout of MTCH2 inhibited tumor progression and metastasis by inducing ferroptosis. Furthermore, MTCH2 knockout (MTCH2^cKO^) mice exhibited suppression of intestinal tumorigenesis induced by AOM/DSS. In addition, our study revealed that knockout of MTCH2 enhanced the efficacy of sorafenib treatment and inhibited the development of liver metastasis. Therefore, these findings suggested that MTCH2 has potential as a predictive factor for CRC prognosis and an indicator for CRC treatment targeting ferroptosis.

## 2 Results

### 2.1 MTCH2 Overexpression in CRC Predicts Poor Prognosis

**Figure 1:**
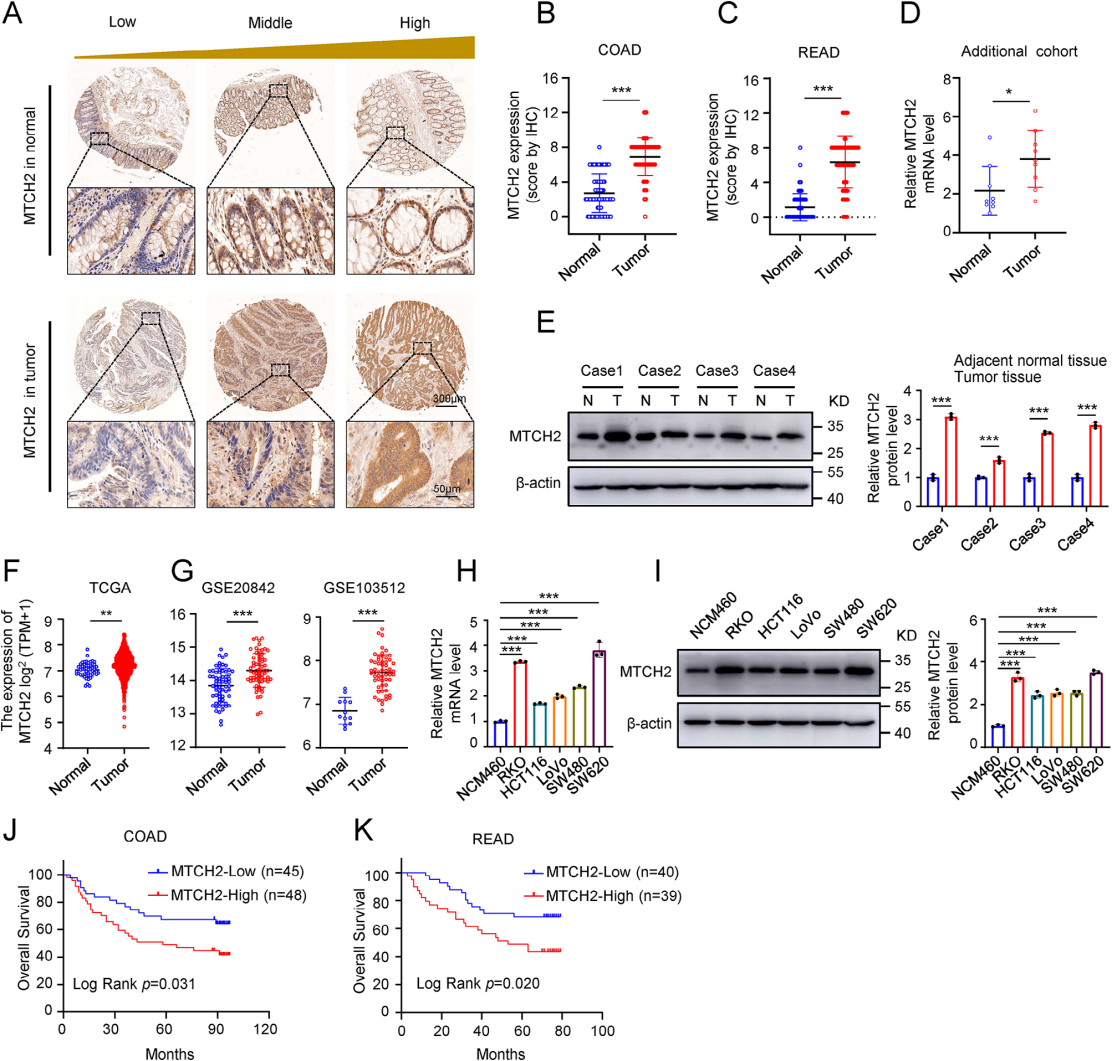
Upregulation of MTCH2 in CRC is associated with poor prognosis. A) Representative immunohistochemistry images with low, medium, and high MTCH2 expression scores in CRC and normal tissues (*n* = 172). Scale bars, 300 µm and 50 µm. B,C) Comparison of MTCH2 IHC expression scores in colon adenocarcinoma (COAD; B) or rectum adenocarcinoma (READ; C) tissues with normal tissues. D,E) Quantification of qRT‐PCR assay of MTCH2 mRNA levels in nine paired CRC samples (D) and Western blot of MTCH2 protein levels in four paired CRC samples (E). F,G) Analysis of MTCH2 expression in CRC tissues from the TCGA (F) and GEO (G) databases. H) Relative mRNA expression levels of MTCH2 in a normal human colon mucosal epithelial cell line (NCM460) and five human CRC cell lines. I) Western blot of MTCH2 expression and quantification of relative protein expression levels (bar graph) in the same cell lines. J,K) Kaplan–Meier curves of overall survival based on MTCH2 IHC staining in COAD (J) and READ (K) samples. Statistics were performed using an unpaired Student's *t*‐test. Data are presented as the means ± SD of at least three independent experiments. *P < 0.05, **P < 0.01, ***P < 0.001.

To determine the clinical significance of MTCH2 in CRC patients, we performed immunohistochemical (IHC) staining on CRC tissue arrays using an anti‐MTCH2 antibody, whose specificity was validated in MTCH2 knockout RKO cells (Figure S1, Supporting Information). IHC‐based grading was used to divide the 172 CRC patients into three expression groups: low (score 0–3), middle (score 4–6), and high (score 7–12) (**Figure** **1**A). MTCH2 staining was obviously higher in both colon adenocarcinoma (COAD) and rectum adenocarcinoma (READ) tissues than in normal tissues (Figure 1B,C).

Quantitative real‐time PCR (qRT‐PCR) analysis further revealed relatively high expression of MTCH2 in nine CRC tissue samples compared with that in paired normal tissues (Figure 1D, Table S1, Supporting Information). Next, a Western blot of four paired CRC and adjacent normal tissues showed upregulation of MTCH2 in CRC tissues (Figure 1E, Table S2, Supporting Information). To validate our findings, we evaluated MTCH2 expression levels in CRC and normal tissue samples from two databases: The cancer genome Atlas (TCGA) and gene expression omnibus (GEO) (GSE20842 and GSE103512). Consistent with our observation, these datasets showed significantly upregulated MTCH2 expression in CRC tissues (Figure 1F,G). We also examined MTCH2 expression in multiple cell lines, which revealed higher MTCH2 mRNA and protein levels in the human CRC cell lines RKO, HCT116, LoVo, SW480, and SW620 than in the normal human colon mucosal epithelial cell line NCM460 (Figure 1H,I).

To investigate the relationship between MTCH2 expression and clinicopathological characteristics of CRC, we first divided the 172 CRC patients into MTCH2‐high (87 cases) and MTCH2‐low (85 cases) expression groups based on IHC scores (**Table** **1**). We found that MTCH2 expression was significantly associated with depth of invasion (*P* = 0.022), lymph node metastasis (*P* = 0.019), and tumor‐node‐metastasis (TNM) stage (*P* = 0.029) (Table 1). However, no significant correlations were found with other clinicopathological variables, including age, gender, tumor location, and tumor differentiation (all *P* > 0.05) (Table 1). Collectively, these data suggested that high MTCH2 expression in CRC is related to depth of invasion, increased lymph node metastases, and a higher TNM stage.

**Table 1:** Correlations between MTCH2 expression and clinicopathological characteristics in CRC patients.

Variables	Cases	MTCH2 expression		
		Low	High	*P‐*Value
Total	172	85	87	
Age [years]				0.410
≤60	53	29	24	
>60	119	56	63	
Gender				0.760
Male	88	42	46	
Female	84	43	41	
Tumor location				0.879
Colon	93	45	48	
Rectum	79	40	39	
Tumor size [cm]				0.430
≤4	62	28	34	
>4	110	57	53	
Depths of invasion				0.022
T1/T2	17	13	4	
T3/T4	155	72	83	
Lymph node metastasis				0.019
Negative	106	60	46	
Positive	66	25	41	
TNM stage				0.029
I/II	105	59	46	
III/IV	67	26	41	
Differentiation				0.223
Well	4	3	1	
Moderate	143	73	70	
Poor	25	9	16	

*P*‐values in bold were statistically significant.

In order to probe the prognostic value of MTCH2, we performed Kaplan–Meier analysis of overall survival (OS) in our CRC cohort. This analysis revealed that MTCH2‐high tumors led to a worse prognosis than MTCH2‐low tumors in patients with both COAD (*P* = 0.031) and READ (*P* = 0.020) (Figure 1J,K). Next, we evaluated the prognostic value of MTCH2 using univariate and multivariate Cox regression analyses. The univariate analysis revealed that MTCH2 expression was a significant predictor of OS (hazard ratio [HR]: 2.315; 95% confidence interval [CI]: 1.461–3.668; *P* < 0.001; **Table** **2**). Furthermore, depth of invasion (*P* = 0.048), lymph node metastasis (*P* < 0.001), TNM stage (*P* < 0.001), and tumor differentiation (*P* < 0.001) were found to be significant variables (Table S2, Supporting Information). The multivariate survival analysis also indicated MTCH2 expression as an independent prognostic factor for poor survival in patients with CRC (HR: 1.973; 95% CI: 1.218–3.196; *P* = 0.006; Table 2).

**Table 2:** Univariate and multivariate analysis of overall survival in CRC patients.

Variables		Univariate			Multivariate	
	HR	95%CI	*P*‐Value	HR	95%CI	*P*‐Value
Age (>60 years vs ≤60 years)	1.159	0.710–1.894	0.555			
Gender (Female vs Male)	0.908	0.586–1.409	0.668			
Tumor location (Rectum vs Colon)	0.934	0.598–1.460	0.764			
Tumor size (>4 vs ≤4 cm)	1.267	0.790–2.033	0.327			
Depth of invasion (T3/T4 vs T1/T2)	3.196	1.008–10.134	0.048	1.775	0.538–5.855	0.346
Lymph node metastasis (N1/N2 vs N0)	2.220	1.431–3.445	<0.001	0.310	0.041–2.358	0.258
TNM stage (III/IV vs I/II)	2.545	1.628–3.979	<0.001	5.846	0.777–43.987	0.086
Tumor differentiation (Poor vs Well/Moderate)	2.792	1.630–4.781	<0.001	2.583	1.498–4.455	0.001
MTCH2 expression (High vs Low)	2.315	1.461–3.668	<0.001	1.973	1.218–3.196	0.006

*HR* hazard ratio, *CI* confidence interval, and *P* values in bold were statistically significant.

### 2.2 MTCH2 Expression Regulates the Proliferation and Metastasis of CRC Cells

**Figure 2:**
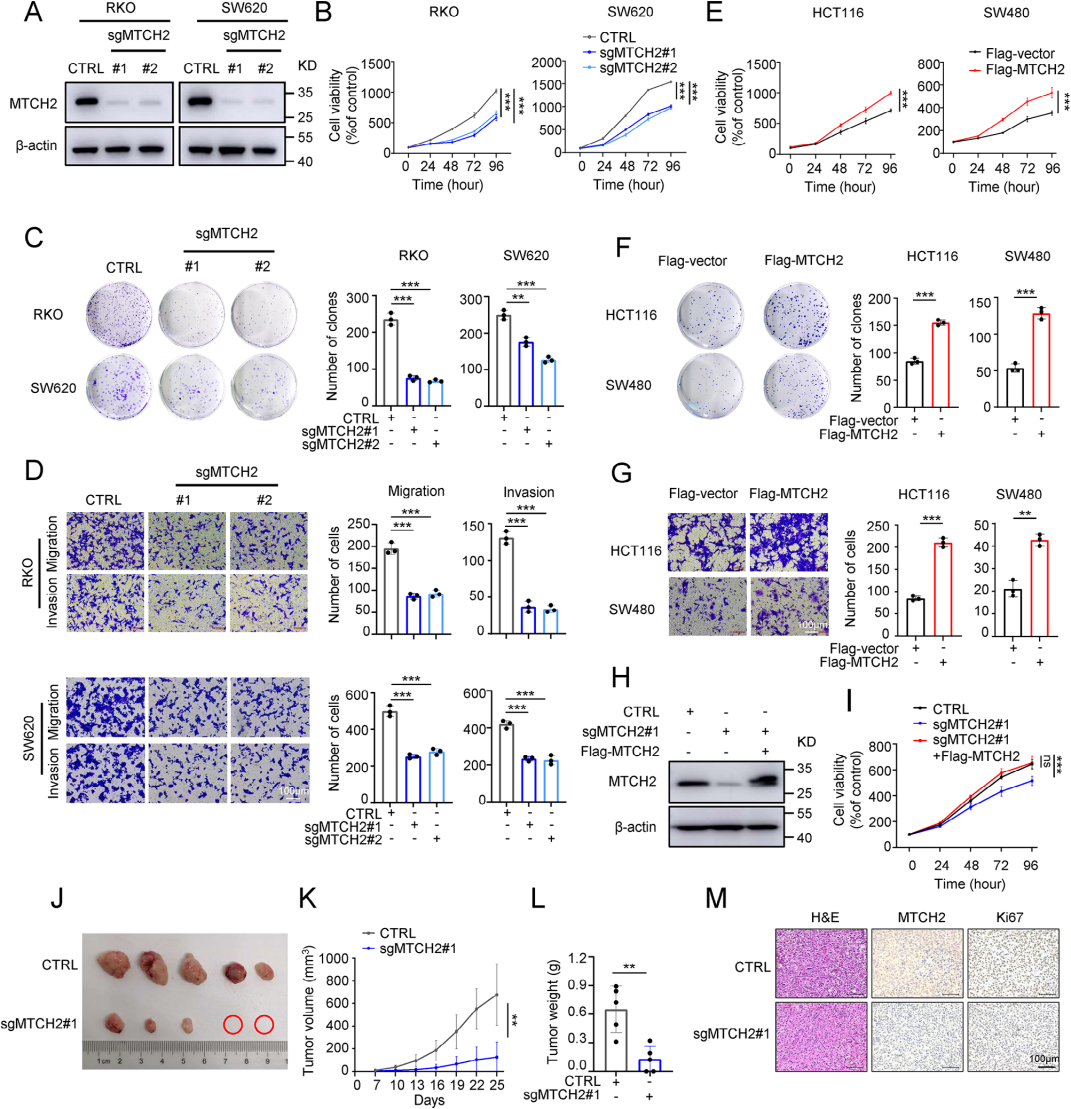
MTCH2 mediates the proliferation, migration, and invasion of CRC cells. A) Western blot assay showing the efficiency of CRISPR/Cas9‐mediated knockout of MTCH2 by transfection with sgMTCH2#1 and #2 in RKO and SW620 cells. B–D) CCK‐8 (B), clonogenic assays (C), and Transwell assays (D) were performed to assess the cell proliferative and metastatic ability of MTCH2 knockout CRC cells. Bar graphs display quantification of colonies (C) and migratory cells (D), respectively. Scale bar, 100 µm. E–G) CCK‐8 (E), clonogenic assays (F), and Transwell assays (G) were performed to evaluate cell growth and metastatic ability in MTCH2‐overexpressing cells. Bar graphs display quantification of colonies (F) and migratory cells (G), respectively. Scale bar, 100 µm. H,I) Western blot analysis of MTCH2 levels (H) and CCK‐8 viability assay (I) of HCT116 cells transfected with sgMTCH2#1 and Flag‐MTCH2 rescue. J) Images of resected mouse tumors after subcutaneous injection with RKO cells stably transfected with sgMTCH2#1 (*n* = 5 per group). Red circles indicate no tumor formation was observed. K) Tumor growth curves of mice injected with wild‐type or MTCH2 knockout RKO cells. L) Mean weight (±SD) of the resected tumors in the two groups. M) Representative histological images of xenografted tumors with hematoxylin and eosin (H&E) and IHC staining for MTCH2 and Ki67. Scale bar, 100 µm. Two‐tailed Student's *t*‐test and one‐way ANOVA with Tukey's multiple comparisons test were used for statistical analysis. The results shown indicate the means ± SD of at least three independent replicates. *P < 0.05, **P < 0.01, ***P < 0.001; ns, not significant.

To investigate the role of MTCH2 in CRC progression, we generated normal colon epithelial NCM460 cells and CRC cells with MTCH2 knockout and overexpression using two single‐guide RNAs (sgRNAs; sgMTCH2#1, sgMTCH2#2) and an expression plasmid (Flag‐MTCH2), respectively. The efficiency of knockout and overexpression was confirmed by Western blot in these cell lines (**Figure** **2**A; Figure S2A,B, Supporting Information). Next, we evaluated the effects of MTCH2 silencing on cell growth and metastasis. While MTCH2 had no noticeable effect on the cell viability of the NCM460 cells (Figure S2C, Supporting Information), its deficiency decreased the proliferation and colony formation of CRC cells (Figure 2B,C). The sgMTCH2‐mediated inhibition of cell proliferation was affirmed by EdU (5‐ethynyl‐2′‐deoxyuridine) staining of tumor cells, which was significantly decreased in MTCH2 knockout cells (Figure S2D, Supporting Information). The cell migration capacity was assessed using Transwell assays, which showed that MTCH2 depletion inhibited the migration and invasion of both CRC cell lines (Figure 2D).

Subsequently, we found that MTCH2 overexpression enhanced CRC cell proliferation, migration, and invasion (Figure 2E–G). Overexpression of MTCH2 protein in cells with MTCH2 knockout cells was verified using Western blot (Figure 2H). The results showed that MTCH2 overexpression relieved the inhibition of HCT116 cell proliferation and migration caused by MTCH2 knockout (Figure 2I; Figure S2E,F, Supporting Information).

The function of MTCH2 was further evaluated in CRC xenografts with MTCH2 knockout, which indicated that suppression of MTCH2 inhibited tumor growth, as reflected by reductions in tumor size, weight, and volume (Figure 2J–L). Furthermore, MTCH2 knockout decreased the expression of Ki67, a biomarker of tumor proliferation in vivo (Figure 2M). Altogether, these results demonstrated that MTCH2 plays an important role in promoting CRC progression.

### 2.3 MTCH2 Knockout Promotes Ferroptosis in CRC Cells

**Figure 3:**
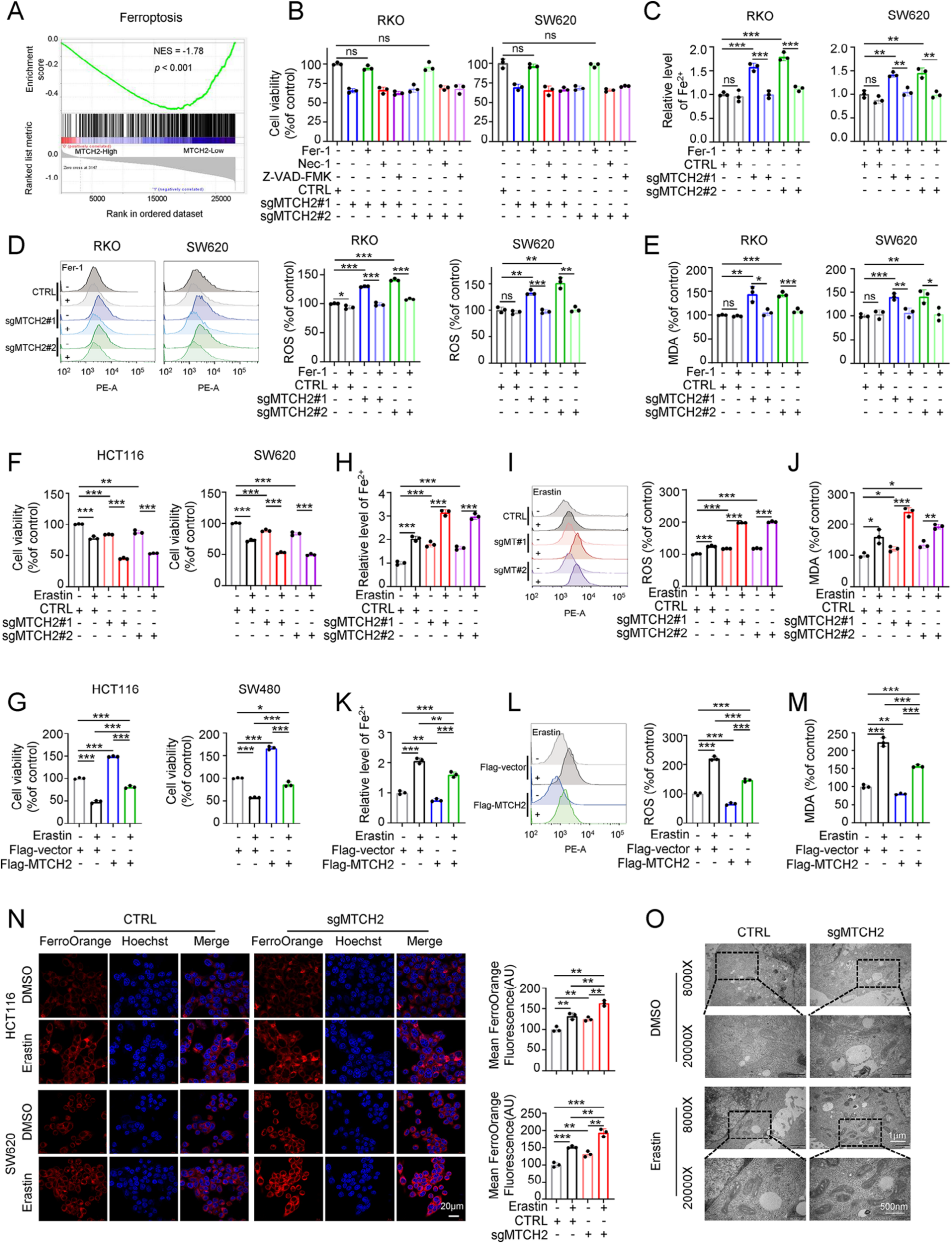
MTCH2 depletion induces ferroptosis in CRC cell lines. A) Gene set enrichment analysis (GSEA) of ferroptosis signaling based on differentially expressed genes related to MTCH2 expression in CRC data from The Cancer Genome Atlas. B) CCK‐8 viability assays in sgRNA control and sgMTCH2 RKO and SW620 cells treated with Fer‐1 (1 µm), Nec‐1 (2 µm), or Z‐VAD‐FMK (20 µm). C–E) Levels of Fe^2+^ (C), ROS (D), and MDA (E) in sgRNA control and sgMTCH2 RKO and SW620 cells treated with Fer‐1 (1 µm). F,G) CCK‐8 viability assays of MTCH2‐knockout (F) or MTCH2‐overexpressing (G) CRC cells treated with erastin. H–J) Levels of Fe^2+^ (H), ROS (I), and MDA (J) in sgRNA control or sgMTCH2 HCT116 cells treated with erastin. K–M) Levels of Fe^2+^ (K), ROS (L), and MDA (M) in MTCH2‐overexpressing HCT116 cells treated with erastin. N) Confocal microscopy images of HCT116 and SW620 cells showing the effect of sgMTCH2#1 transfection with or without erastin treatment (20 µm) on cellular Fe^2+^ levels detected using FerroOrange. Bar graphs represent the quantification of mean fluorescent intensity. Scale bar, 20 µm. O) Transmission electron microscopy images of mitochondrial morphology in MTCH2 knockout HCT116 cells with the treatment of erastin. Scale bars, 1 µm and 500 nm. Two‐way ANOVA with Tukey's multiple comparisons test was used for statistical analysis. The results shown indicate the means ± SD of at least three independent replicates. *P < 0.05, **P < 0.01, ***P < 0.001; ns, not significant. “sgMT” refers to “sgMTCH2”.

To better understand the effects of MTCH2 on the growth and metastasis of CRC cells, we performed Gene Set Enrichment Analysis (GSEA) using TCGA data. The bioinformatics analysis showed that MTCH2 is implicated in ferroptosis (**Figure** **3**A, Table S3, Supporting Information). However, there was no significant association of MTCH2 with necrosis, apoptosis, or autophagy (Figure S3A, Supporting Information). To verify this result, we used cell death inhibitors to block necroptosis, ferroptosis, or apoptosis in RKO and SW620 cells. Consistent with the GSEA results, only the ferroptosis inhibitor (Fer‐1) rescued the inhibitory effect of MTCH2 knockout on CRC cell growth (Figure 3B). These findings indicated that the tumor‐suppressive effect of the MTCH2 knockout is related to its involvement in ferroptosis but not necroptosis or apoptosis.

Levels of ferrous ion (Fe^2+^), ROS, malondialdehyde (MDA), and lipid peroxidation are the main indicators of ferroptosis. We found that MTCH2 inhibition markedly increased Fe^2+^, ROS, MDA, and lipid peroxidation levels of CRC cells, which could be reversed by treatment with Fer‐1 (Figure 3C–E; Figure S3B,C, Supporting Information). These results suggested that MTCH2 deficiency may inhibit the proliferation and metastasis of CRC cells by inducing ferroptosis.

Erastin is an activator of ferroptosis, resulting in a reduction of cell viability.^[^ ^27^ ^]^ As expected, erastin inhibited CRC cell proliferation in a dosage‐dependent manner (Figure S3D, Supporting Information). This effect was enhanced by the knockout of MTCH2 (Figure 3F). However, MTCH2 overexpression significantly decreased erastin‐induced inhibition of CRC cell viability (Figure 3G). Further results showed that MTCH2 silencing facilitated the erastin‐induced increases in Fe^2+^, ROS, MDA, and lipid peroxidation levels in HCT116 and SW620 cells (Figure 3H–J; Figure S3E–H, Supporting Information), while low levels of Fe^2+^, ROS, MDA, and lipid peroxidation levels were detected in cells overexpressing MTCH2, compared with the control group (Figure 3K–M; Figure S3I–L, Supporting Information). Moreover, overexpression of MTCH2 indeed rescued the sgMTCH2‐induced inhibition of cell viability, with or without erastin treatment (Figure S3M, Supporting Information). Similarly, the increases of Fe^2+^, ROS, and MDA levels induced by MTCH2 knockout, with or without erastin treatment, were reversed by overexpression of MTCH2 (Figure S3N–P, Supporting Information). Therefore, these data suggested that MTCH2 deficiency enhances ferroptosis, with MTCH2 overexpression impairing the sensitivity of these cells to erastin.

Additionally, confocal microscopy using a Fe^2+^ fluorescent probe showed that MTCH2 knockout significantly raised the intracellular Fe^2+^ concentration upon treatment with erastin, as reflected by an increase in the orange fluorescence intensity (Figure 3N). Changes in mitochondrial morphology represent another important characteristic of ferroptosis. The detailed structures obtained by transmission electron microscopy (TEM) showed that depletion of MTCH2 enhanced the effects of erastin, as exhibited by decreased numbers of mitochondria and increased membrane density (Figure 3O). Therefore, these findings suggested that MTCH2 knockout facilitated erastin‐induced ferroptosis in CRC cells.

### 2.4 MTCH2 Knockout Enhances Ferroptosis Dependent on the Level of TFRC

**Figure 4:**
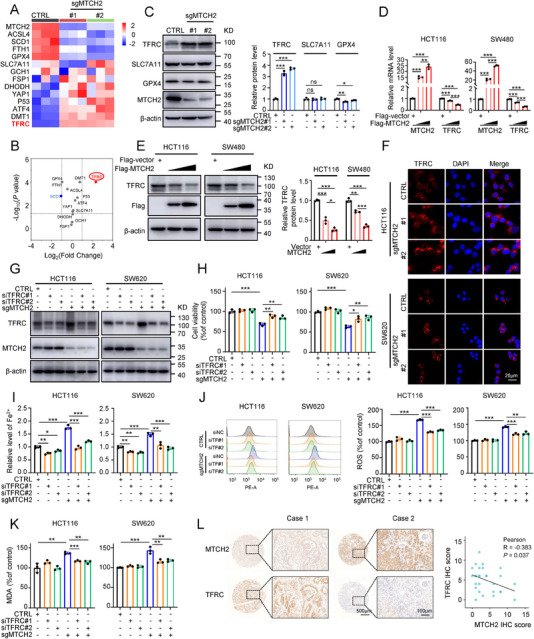
Knockout of MTCH2 promotes ferroptosis through TFRC. A) Heat map of normalized mRNA levels of 13 ferroptosis‐associated genes detected in control or sgMTCH2 HCT116 cells using qRT‐PCR. B) A volcano plot illustrating the fold changes and *P* values of the 13 ferroptosis‐associated genes. C) Levels of TFRC, SLC7A11, GPX4, and MTCH2 were detected and quantified (in the bar graph) in HCT116 cells with loss of MTCH2. D,E) qRT‐PCR (D) and Western blot (E) analyses of TFRC expression in HCT116 and SW620 cells transfected with indicated concentrations of Flag‐MTCH2 plasmid or control plasmid. F) Representative immunofluorescence images of TFRC in HCT116 and SW620 cells with MTCH2 knockout. Scale bar, 20 µm. G–K) The levels of TFRC (G) and cell viability (H), as well as levels of Fe^2+^ (I), ROS (J), and MDA (K), were measured in MTCH2 knockout CRC cell lines transfected with siTFRC. L) Representative IHC images of MTCH2 and TFRC staining of colorectal tumor samples from CRC patients. The graph depicts Pearson correlation analysis based on IHC scores (*n* = 30). Scale bars, 500 and 100 µm. The statistical analysis was calculated by two‐way ANOVA for multiple comparisons, one‐way ANOVA with Tukey's honest difference post hoc test, or Pearson's correlation test. Data present means ± SD from three independent experiments. *P < 0.05, **P < 0.01, ***P < 0.001; ns, not significant.

Next, we explored the mechanism by which MTCH2 regulates ferroptosis. The key molecular mechanisms of ferroptosis involve lipid peroxidation, iron accumulation, antioxidant defense, and transcription factors.^[^ ^28^ ^]^ Therefore, we performed qRT‐PCR to screen for ferroptosis‐associated downstream targets of MTCH2 modulation. The heat map shown in **Figure** **4**A illustrates the detection of 13 ferroptosis‐related genes based on a comparison of normalized mRNA expression in sgRNA control and sgMTCH2#1 and #2 groups of HCT116 cells. Relative mRNA expression levels of the group samples revealed that MTCH2 depletion upregulated TFRC, DMT1, p53, ATF4, and YAP1, and downregulated ACSL4, SCD1, FTH1, and GPX4 (Figure S4A, Supporting Information). A volcano plot and Western blot assays identified TFRC as the top candidate among these ferroptosis‐related genes, including SLC7A11 and GPX4, showing the largest fold‐change difference (Figure 4B,C). Previous studies demonstrated that TFRC increased the intracellular labile iron pool and mediated iron uptake, leading to ferroptosis.^[^ ^29^ ^]^ We found that MTCH2 knockout increased the level of TFRC protein (Figure S4B, Supporting Information), whereas MTCH2 overexpression significantly decreased TFRC at the mRNA and protein levels in a manner dependent on the overexpression plasmid concentration in HCT116 and SW620 cells (Figure 4D,E). Moreover, immunofluorescence staining confirmed that CRC cells with MTCH2 knockout showed increased levels of TFRC compared with control cells (Figure 4F).

Next, we investigated whether TFRC is involved in the regulation of MTCH2‐related ferroptosis via small interfering RNA‐mediated knockdown of TFRC levels in wild‐type and sgMTCH2‐transfected HCT116 and SW620 cells. The efficiencies of the transient transfections were verified by qRT‐PCR and Western blot (Figure 4G; Figure S4C,D, Supporting Information). CCK‐8 assays showed that the MTCH2 knockout‐induced inhibition of cell proliferation was partially reversed by the depletion of TFRC (Figure 4H). Correspondingly, TFRC knockdown prevented the MTCH2 knockout‐induced increases in Fe^2+^, ROS, and MDA (Figure 4I–K). To further elucidate the relationship between MTCH2 and TFRC, we evaluated their expression in 30 CRC tissue samples from patients using IHC. Statistical analysis revealed a negative correlation between the expression levels of MTCH2 and TFRC (Figure 4L). These findings suggested that MTCH2 silencing‐induced ferroptosis in CRC depends on the level of TFRC.

### 2.5 MTCH2 Inhibits TFRC Expression by Stabilizing E2F4 Protein via the Ubiquitin‐Proteasome Pathway

**Figure 5:**
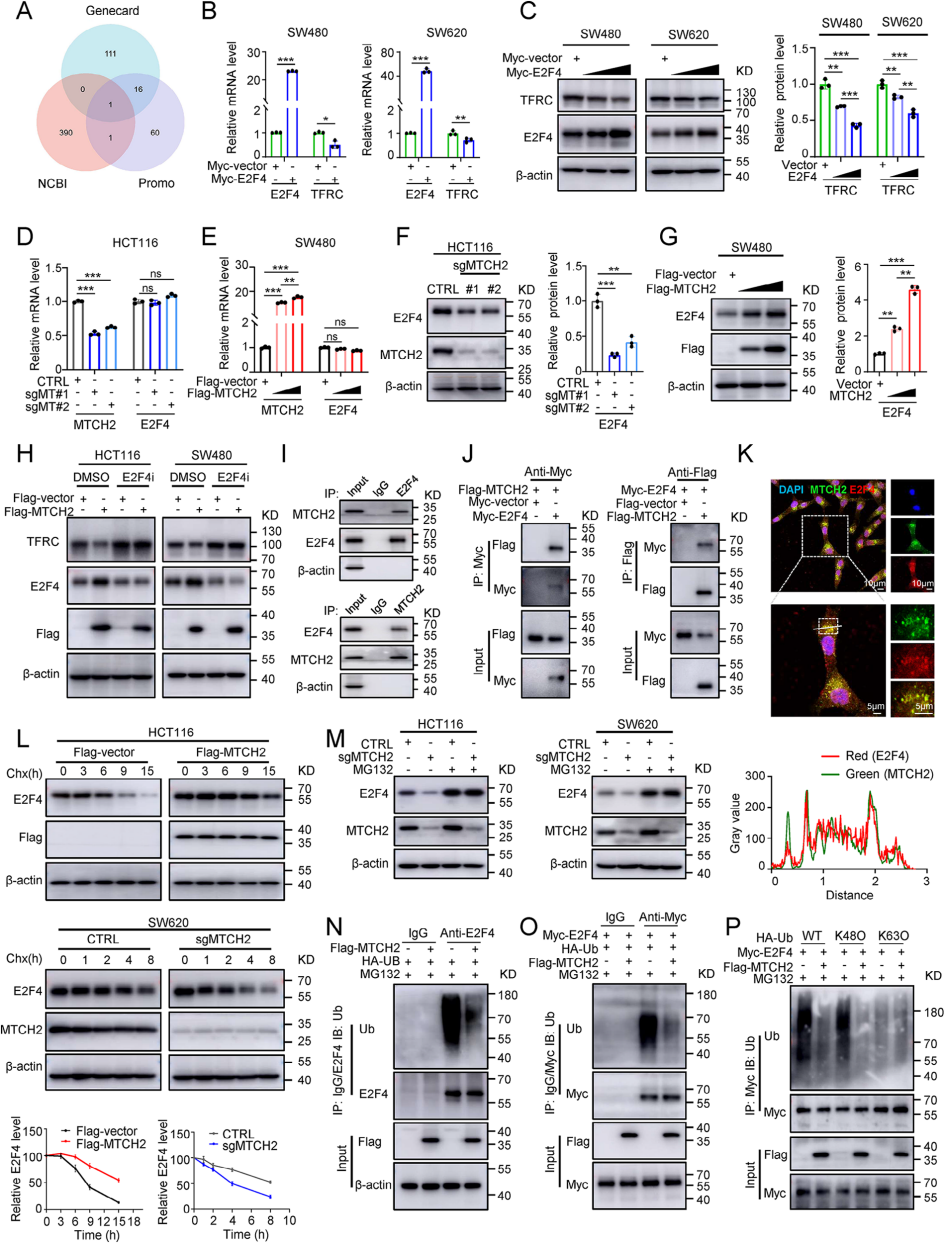
MTCH2 decreases TFRC levels by repressing E2F4 degradation via inhibition of its ubiquitination. A) Venn diagram of the intersection of transcription factors of TFRC predicted to interact with MTCH2 at online databases (Genecard, PROMO, and NCBI). B,C) qRT‐PCR (B) and Western blot (C) analysis of TFRC mRNA and protein expression in SW480 and SW620 cells transfected with the Myc‐vector or Myc‐E2F4 plasmid. D–G) The mRNA (D,E) and protein levels (F,G) of E2F4 and MTCH2 in MTCH2‐knockout HCT116 cells and MTCH2‐overexpressing SW480 cells. H) Western blot analysis showing rescue of TFRC levels in MTCH2‐overexpressing CRC cells treated with or without E2F4 inhibitor (E2F4i) (40 µm). I) Co‐immunoprecipitation (IP) assay for the endogenous interaction between MTCH2 and E2F4 in HCT116 cells. J) Western blot of Co‐IP assays of exogenous MTCH2 and E2F4 using an anti‐Myc antibody to pull down Myc‐E2F4 (left) or anti‐Flag antibody to pull down Flag‐MTCH2 (right) from lysates of HCT116 cells transfected as indicated. Blots were probed using the indicated antibodies. K) Representative pictures of co‐localization with MTCH2 (green) and E2F4 (red) fluorescent proteins were obtained by confocal microscopy. The fluorescence intensity graph (bottom) shows the level of co‐localization, and each line indicates the corresponding fluorescence. Scale bars, 10 and 5 µm. L) Cycloheximide (Chx) chase assays and quantification of E2F4 protein levels in MTCH2‐overexpressing HCT116 (upper panels) and sgMTCH2 SW620 cells (lower panels) for the indicated times. M) Western blot of E2F4 in lysates of sgMTCH2 HCT116 and SW620 cells treated with MG132 (10 µm). N,O) MTCH2 overexpression inhibited endogenous (M) and exogenous (N) ubiquitination of E2F4. An endogenous ubiquitination assay was performed in HCT116 cells. Exogenous ubiquitination assay was conducted following IP assay with an anti‐Myc antibody in HEK293T cells transfected with Myc‐E2F4, HA‐Ub, Flag‐vector, and Flag‐MTCH2 plasmids for 24 h and subsequently treated with or without MG132 (10 µm) for 8 h. The blot was probed with the indicated antibodies. P) The K48‐ and K63‐linked ubiquitination patterns on E2F4 were examined using HCT116 cells transfected with indicated constructs, including HA‐Ub or its mutants containing only one lysine at either K48 or K63. Two‐tailed Student's *t*‐test and one‐way ANOVA with Tukey's honest difference post hoc tests were used for statistical analysis. The results shown indicate the means ± SD of at least three independent replicates. *P < 0.05, **P < 0.01, ***P < 0.001; ns, not significant.

We next sought to determine the mechanism by which MTCH2 regulates TFRC. Our findings thus far suggested that MTCH2 regulated TFRC expression at both the mRNA and protein levels (Figure 4C–E). However, co‐immunoprecipitation experiments indicated that there was no physical interaction between MTCH2 and TFRC (Figure S5A,B, Supporting Information), suggesting that TFRC might be regulated by MTCH2 at the transcriptional level. However, there are no reports of MTCH2 as a transcriptional factor in the literature. Therefore, we determined whether there are other factors involved. We predicted the potential transcription factors regulating TFRC expression using the GeneCard (https://www.genecards.org) and PROMO(https://alggen.lsi.upc.es/cgi‐bin/promo_v3/promo/promoinit.cgi?dirDB = TF_8.3) databases. Subsequently, an intersection was extracted between these potential transcription factors of TFRC and the MTCH2‐interacting proteins provided from the NCBI database (https://www.ncbi.nlm.nih.gov/gene/23788). Interestingly, E2F4, a speculative MTCH2‐interacting protein, was identified as having the potential to directly regulate TFRC (**Figure** **5**A).

Indeed, E2F4 overexpression significantly downregulated the mRNA and protein levels of TFRC in SW480 and SW620 cells (Figure 5B,C). Regarding the association between MTCH2 and E2F4, qRT‐PCR results suggested that knockout or overexpression of MTCH2 had little effect on E2F4 mRNA levels (Figure 5D,E). At the protein level, MTCH2 knockout significantly decreased E2F4 expression (Figure 5F), whereas MTCH2 overexpression significantly increased E2F4 expression in a plasmid dose‐dependent manner (Figure 5G). Moreover, exogenous supplementation of MTCH2 rescued the E2F4 downregulation caused by MTCH2 depletion (Figure S5C, Supporting Information). Additionally, treatment with an E2F4 inhibitor (E2F4i) blocked the regulation of TFRC induced by MTCH2 overexpression (Figure 5H; Figure S5D, Supporting Information).

Next, we investigated the underlying mechanism by which MTCH2 positively regulates E2F4 expression. Previous studies indicated that MTCH2 may serve as a key molecule in the ubiquitin‐proteasome pathway, mediating protein ubiquitination.^[^ ^30^ ^]^ Thus, we explored the possible interaction between MTCH2 and E2F4. We found that MTCH2 can directly interact with E2F4 in both exogenous and endogenous cell systems by co‐immunoprecipitation (Figure 5I,J). Subsequently, confocal microscopy experiments further demonstrated the colocalization of MTCH2 with E2F4 in the cytoplasm of HCT116 cells (Figure 5K; Figure S5E, Supporting Information).

Since MTCH2 is an outer‐mitochondrial membrane protein, we determined whether MTCH2 and E2F4 colocalize in the mitochondria. Co‐immunofluorescence staining was performed using antibodies against MTCH2, E2F4, and the mitochondrial marker TOM20. The results showed that endogenous MTCH2 is localized in the mitochondria (Figure S5F, Supporting Information). Meanwhile, E2F4 was found to colocalize with both MTCH2 and TOM20 (Figure S5F, Supporting Information). Similarly, cytoplasmic fractionation analysis followed by Western blot confirmed that MTCH2 was predominantly localized in the mitochondrial fraction, whereas E2F4 was mainly cytosolic, but a small fraction of E2F4 was detected in the mitochondrial fraction (Figure S5G, Supporting Information). MTCH2 deficiency reduced E2F4 expression in cell mitochondria, and overexpression of MTCH2 produced the opposite result (Figure S5G, Supporting Information). Furthermore, to determine whether E2F4 could physically interact with MTCH2 when the outer‐mitochondrial membrane is intact, we performed formaldehyde cross‐linking in intact cells prior to mitochondrial isolation, followed by Co‐IP. The result demonstrated a physical interaction between MTCH2 and E2F4 in mitochondrial fractions (Figure S5H, Supporting Information).

Given that E2F4 can be degraded through the ubiquitin‐proteasome pathway and MTCH2 plays a role in ubiquitination,^[^ ^30^, ^31^ ^]^ we sought to determine whether MTCH2 regulates E2F4 ubiquitination. In CRC cells pre‐treated with cycloheximide (CHX), an inhibitor of protein synthesis, we detected changes in E2F4 protein levels at several different time points. Notably, overexpression and silencing of MTCH2 significantly increased and decreased the half‐life of E2F4 protein, respectively, in these cells (Figure 5L). Moreover, incubation of HCT116 cells with the proteasome inhibitor MG132 prevented the degradation of E2F4 protein induced by MTCH2 knockout (Figure 5M).

In addition, a ubiquitination‐based immunoprecipitation assay was carried out to investigate the influence of MTCH2 on E2F4 ubiquitination. The results showed that overexpression of MTCH2 attenuated the ubiquitination of endogenous and exogenous E2F4 ubiquitination in HEK293T cells treated with MG132 (Figure 5N,O). We further found that MTCH2‐mediated E2F4 ubiquitination could only be detected in the presence of the K48‐Ub plasmid (Figure 5P). Collectively, these results suggested that MTCH2 stabilizes E2F4 protein by interfering with the K48‐linked ubiquitin‐proteasome protein degradation pathway in CRC cells.

### 2.6 MTCH2 Represses TFRC Expression Through E2F4‐Mediated Transcription Regulation

**Figure 6:**
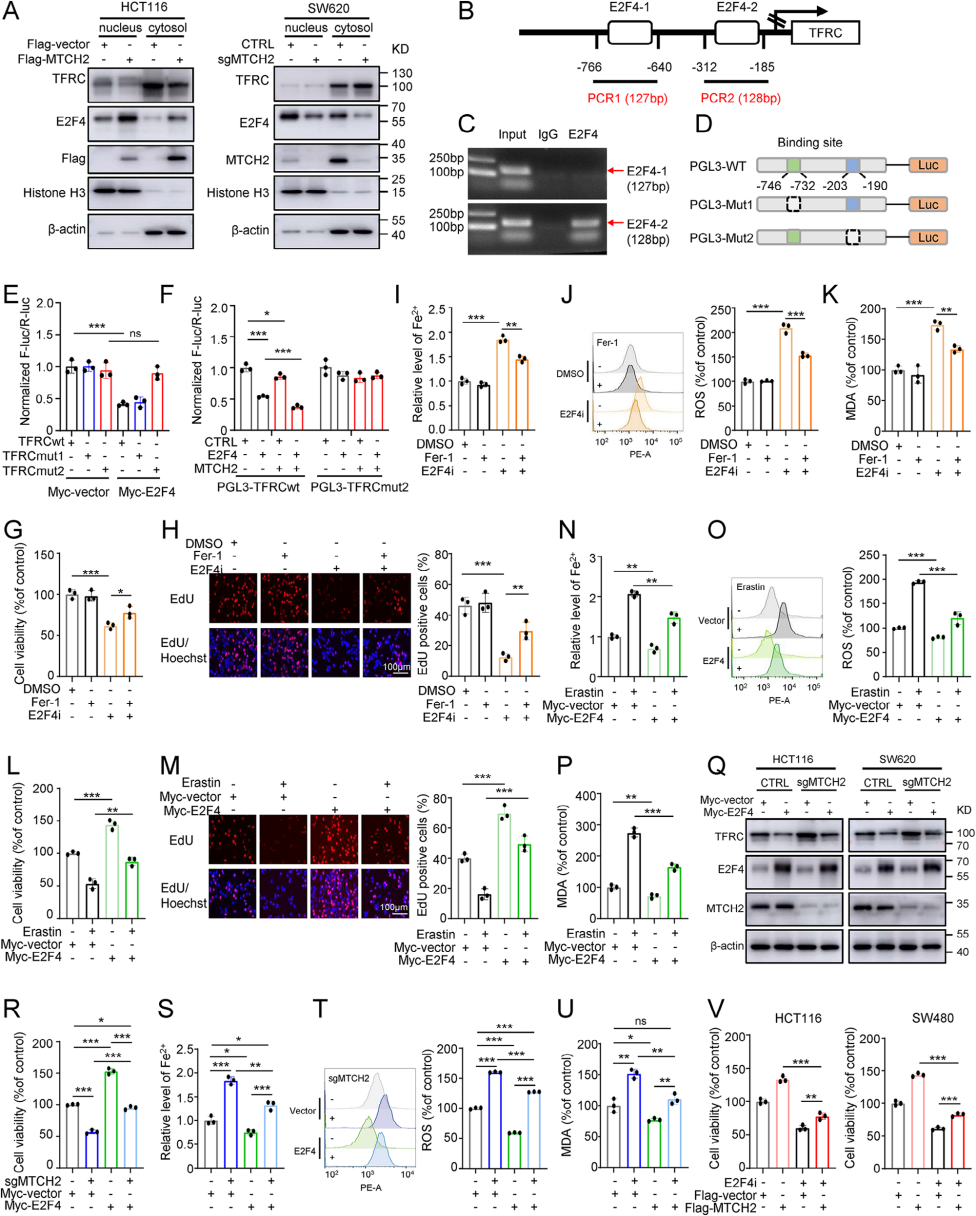
MTCH2 enhances E2F4‐modulated repression of TFRC transcription. A) Distributions of TFRC and E2F4 in cytoplasmic and nuclear fractions of MTCH2‐overexpressing HCT116 cells (left panels) and sgMTCH2 SW620 cells (right panels). β‐actin and histone H3 were used as cytoplasmic and nuclear markers, respectively. B) Two predicted binding sites for E2F4 in the TFRC promoter. C) Chromatin immunoprecipitation assay of the E2F4 binding sites in the promoter region of TFRC in HCT116 cells. D) Schematic representation of wild‐type (PGL3‐WT) and mutated (PGL3‐Mut1, PGL3‐Mut2) TFRC promoter luciferase reporter constructs. E,F) Luciferase reporter assays showing the influence of Myc‐E2F4 expression (E) and MTCH2 overexpression (F) on wild‐type (TFRCwt) and mutated (TFRCmut1 and TFRCmut2) promoter expression in HCT116 cells. The relative luciferase activity was determined by calculating the ratio of firefly luciferase activity to Renilla luciferase activity. G–K) Cell viability (G), EdU assays (H), the levels of Fe^2+^ (I), ROS (J), and MDA (K) of HCT116 cells treated with an E2F4i (40 µm) and Fer‐1 (1 µm). Scale bar, 100 µm. L–P) Cell viability (L), EdU assays (M), the levels of Fe^2+^ (N), ROS (O), and MDA (P) of E2F4‐overexpressing HCT116 cells treated with or without erastin (20 µm). Scale bar, 100 µm. Q) Western blot analysis showing the rescue of TFRC levels in sgMTCH2 CRC cells transfected with Myc‐E2F4 plasmid. R–U) Cell viability (R), the levels of Fe^2+^ (S), ROS (T), and MDA (U) of sgMTCH2 HCT116 cells transfected with Myc‐E2F4 plasmid. V) CCK‐8 viability assay of MTCH2‐overexpressing HCT116 and SW480 cells treated with E2F4i (40 µm). Two‐way ANOVA was used for statistical analysis. The results shown indicate the means ± SD of at least three independent replicates. *P < 0.05, **P < 0.01, ***P < 0.001; ns, not significant.

To further explore how E2F4, which mainly functions as a transcription factor,^[^ ^32^ ^]^ regulates TFRC in CRC cells, we first investigated whether the subcellular distributions of E2F4 and TFRC were regulated by MTCH2. Using separate nuclear and cytoplasmic fractions of HCT116 and SW620 cells, we found that Flag‐MTCH2 significantly increased the accumulation of E2F4 in the nucleus and inhibited the level of TFRC in the cytoplasm, while MTCH2 knockout reduced nuclear translocation of E2F4 and increased the cytoplasmic TFRC level (**Figure** **6**A).

To further define the role of E2F4 in TFRC transcription, we predicted two putative E2F4 binding sites (E2F4‐1 and E2F4‐2) within the TFRC promoter region using Jaspar (http://jaspar.genereg.net/) (Figure 6B). Next, a chromatin immunoprecipitation assay was conducted to evaluate promoter binding, which demonstrated that E2F4 bound to the E2F4‐2 region (−312 to −185 bp), but not to the E2F4‐1 region (−766 to −640 bp) (Figure 6C). To determine the precise binding site, two TFRC promoter‐mutant plasmids (TFRCmut1 and TFRCmut2) were constructed for dual‐luciferase activity assays (Figure 6D). As expected, E2F4 overexpression decreased the luciferase activities of the wild‐type (TFRCwt) and TFRCmut1 (−746 to −732 bp) promoters, while TFRCmut2 (−203 to −190 bp) had no obvious promoter activity (Figure 6E). Moreover, overexpression of MTCH2 further enhanced the repressive transcriptional activity of E2F4 at the TFRCwt promoter, but not at TFRCmut2 (Figure 6F). Collectively, these findings demonstrated that E2F4 can inhibit the transcriptional activity of the TFRC promoter by directly binding to a specific site (−203 to −190 bp) and that MTCH2 combined with E2F4 could synergistically suppress TFRC transcription.

Although E2F4 has been reported to enhance tumor cell proliferation,^[^ ^33^ ^]^ there is no literature addressing whether the effects of E2F4 in cancer growth are associated with ferroptosis. Thus, to determine the role of E2F4 in ferroptosis, HCT116 cells overexpressing or inhibiting E2F4 were treated with ferroptosis inhibitor Fer‐1 or ferroptosis inducer erastin. The results showed that Fer‐1 could rescue the inhibitory effect on cell proliferation (Figure 6G,H) and reverse the elevations in Fe^2+^, ROS, and MDA levels that were induced by E2F4 inhibitors (E2F4i) (Figure 6I–K). Meanwhile, erastin partially reversed the effect of E2F4 overexpression on cell viability (Figure 6L,M), as well as the levels of Fe^2+^, ROS, and MDA (Figure 6N–P). These results indicated that E2F4 is implicated in ferroptosis.

Next, we sought to determine whether E2F4 is involved in MTCH2 knockout‐mediated ferroptosis. Western blot analysis showed that overexpression of E2F4 weakened the upregulation of TFRC levels caused by MTCH2 deficiency (Figure 6Q). Correspondingly, the upregulation of E2F4 reversed the effect of MTCH2 knockout on cell viability (Figure 6R), as well as the levels of Fe^2+^, ROS, and MDA (Figure 6S–U). Moreover, E2F4i compromised the hyperproliferation of CRC cells elicited by Flag‐MTCH2 overexpression (Figure 6V). These findings suggest that MTCH2 depletion‐induced ferroptosis is dependent on E2F4.

### 2.7 Conditional MTCH2 Knockout in the Intestine Suppresses AOM/DSS‐Induced CRC In Vivo

**Figure 7:**
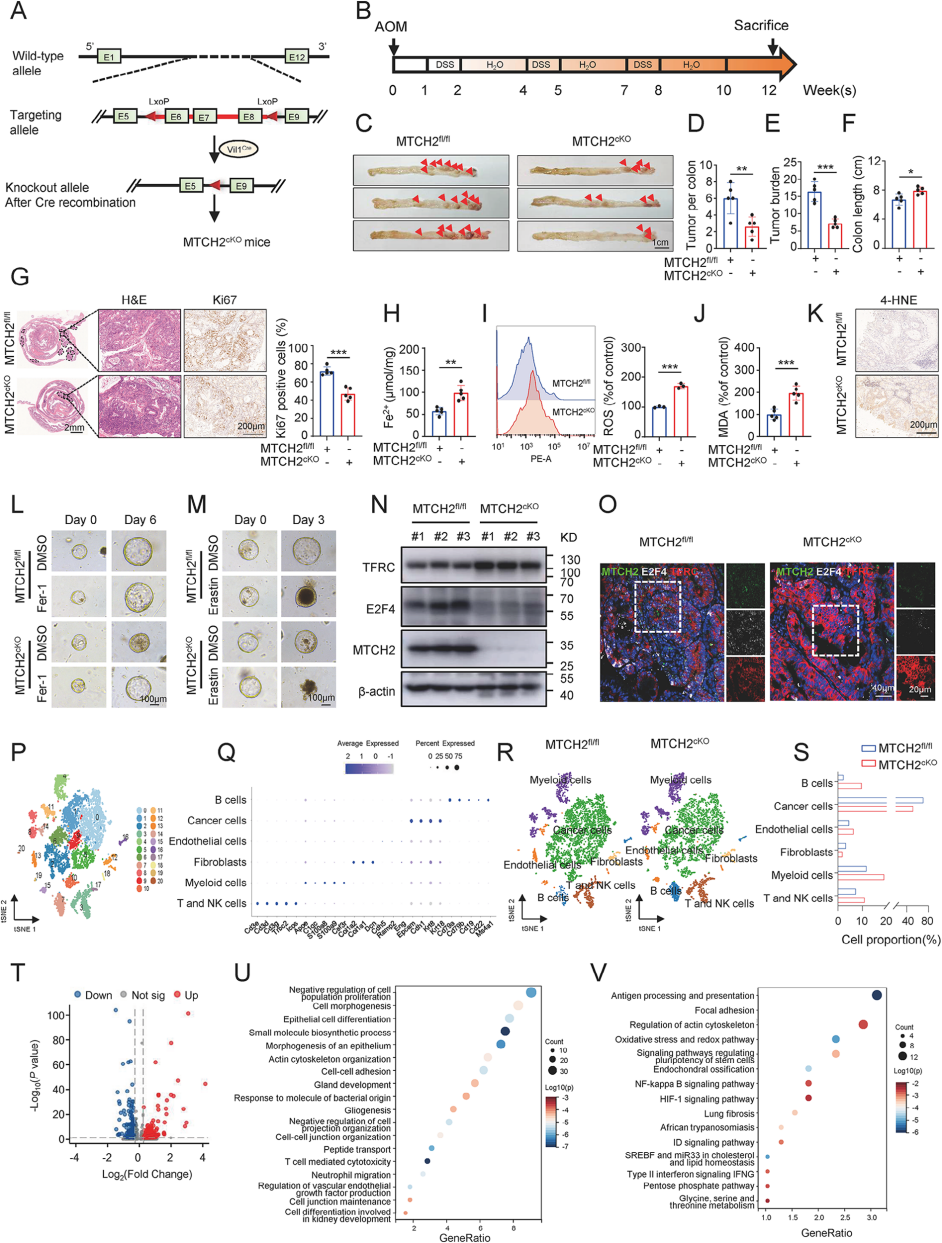
Conditional knockout of MTCH2 inhibits AOM/DSS‐induced CRC in mice. A) Schematic diagram of the construction of intestine‐specific MTCH2 knockout (MTCH2^cKO^) mice. B) Workflow of azoxymethane (AOM)/dextran sodium sulfate (DSS) induction of colitis‐associated CRC model mice. C) Representative images of colorectal tumors (red arrowheads) in control MTCH2^fl/fl^ mice and MTCH2^cKO^ mice. Scale bar, 1 cm. D–F) Tumor number (D) and tumor burden (E) per colon, and colon length (F) in the two groups (*n* = 5 per group). G) H&E and IHC staining of Ki67 in colorectal tissues from MTCH2^fl/fl^ and MTCH2^cKO^ mice. Quantification of Ki67 expression is shown in the bar graph. Scale bars, 2 mm and 200 µm. H–K) Levels of Fe^2+^ (H), ROS (I), MDA (J), and 4‐HNE (K) in the two groups. Scale bar, 200 µm. L,M) Representative image of MTCH2^fl/fl^ and MTCH2^cKO^ mouse tumor organoids exposed to Fer‐1 (5 µm) (L) or Erastin (4 µm) (M). Scale bar, 100 µm. N) Western blot of TFRC, E2F4, and MTCH2 in colorectal tumors of the two groups of mice (*n *= 3 per group). O) Representative immunofluorescence images of MTCH2, E2F4, and TFRC in colorectal tumors of the two groups. Green indicates MTCH2, white indicates E2F4, and red indicates TFRC. Scale bars, 40 µm (left column), 20 µm (right column). P) Fresh tumor samples obtained from groups of MTCH2^fl/fl^ and MTCH2^cKO^ mice, respectively, were pooled and subjected to single‐cell RNA sequencing. T‐distributed stochastic neighbor embedding (t‐SNE) visualization of unsupervised segregation of infiltrating cells into 21 clusters. Q) Dot plot showing the expression of cluster marker genes. The color scale represents the average gene expression level. Circle size represents the percentage of cells expressing the gene in each cluster. R,S) Proportions of the six cell clusters in MTCH2^fl/fl^ and MTCH2^cKO^ mice presented as tSNE plots (R) and a bar chart (S). T) Volcano plot of differentially expressed genes (DEGs) in CRC tumor cells between MTCH2^fl/fl^ and MTCH2^cKO^ mice. U,V) Gene ontology (U) and pathway enrichment analyses (V) for the DEGs from tumor cells of mice. Two‐tailed Student's *t*‐test was used for statistical analysis. The results shown indicate the means ± SD of at least three independent replicates. *P < 0.05, **P < 0.01, ***P < 0.001.

To evaluate the role of MTCH2 in colorectal carcinogenesis in vivo, we constructed intestine‐specific MTCH2 knockout mice by crossing MTCH2^fl/fl^ mice with Villin‐Cre mice (**Figure** **7**A). The recombinant genotype was confirmed by PCR amplification of genomic DNA (Figure S6A, Supporting Information), and the knockout efficiency in intestinal cells was verified by qRT‐PCR and Western blot (Figure S6B,C, Supporting Information). To construct the colitis‐associated cancer model, we treated mice with a combination of AOM and DSS (Figure 7B), monitoring changes in body weight and occult blood to evaluate the success of CRC induction. As expected, we observed that mice in MTCH2^fl/fl^ and MTCH2^cKO^ groups experienced repeated weight loss and hematochezia (Figure S6D,E, Supporting Information). Meanwhile, bleeding scores were slightly lower in MTCH2^cKO^ mice than in MTCH2^fl/fl^ mice (Figure S6E, Supporting Information). After 12 weeks of AOM/DSS treatment, the mice were sacrificed for analysis of tumor formation and measurement of colorectal length. Compared with MTCH2^fl/fl^ mice, MTCH2^cKO^ mice exhibited marked reductions in tumor number and tumor burden (Figure 7C–E), as well as a longer colorectal length (Figure 7F; Figure S6F, Supporting Information). Histological analysis of colorectal tissues using H&E staining confirmed CRC formation (Figure 7G), while IHC confirmed that the proportion of Ki67‐positive cells was decreased in MTCH2^cKO^ mice compared with that in MTCH2^fl/fl^ mice (Figure 7G). These results indicated that MTCH2 deficiency suppressed tumorigenesis in CRC induced by AOM and DSS.

To determine the effect of MTCH2 on ferroptosis, we examined the indexes of ferroptosis in CRC tissues from these mice. The levels of Fe^2+^, ROS, MDA, and 4‐HNE were elevated in MTCH2^cKO^ mice compared with those in MTCH2^fl/fl^ mice (Figure 7H–K). Furthermore, Fer‐1 rescued the inhibition of mouse tumor organoid growth that had been induced by MTCH2 deficiency, and erastin markedly enhanced the MTCH2 depletion‐induced inhibition of proliferation (Figure 7L,M; Figure S6G,H, Supporting Information). To confirm the molecular mechanism of ferroptosis regulation by MTCH2 in vivo, we assessed the expression levels of TFRC and E2F4 using Western blot, qRT‐PCR, and IHC. Similar to the results in CRC cell lines, TFRC levels were notably upregulated and E2F4 levels were downregulated in MTCH2^cKO^ mice (Figure 7N; Figure S6I,J, Supporting Information). These findings were further validated through multiplexed IHC staining (Figure 7O). Additionally, we observed that GPX4 levels were lower, while SLC7A11 expression showed no significant change in the MTCH2^cKO^ group compared with the control (Figure S6K, Supporting Information).

To determine whether MTCH2 affected the inflammation induced by DSS, we treated control and MTCH2 KO NCM460 normal colon epithelial cells with DSS and measured inflammatory markers. Although DSS significantly upregulated IL‐1β and IL‐6 in both groups, no difference was observed between control and KO cells (Figure S6L, Supporting Information). Moreover, we assessed the association between the MTCH2 expression and colitis using public databases. MTCH2 mRNA levels remained unchanged in DSS‐treated colitis mice compared to the untreated control mice (GSE244377, GSE252812, and GSE247433) (Figure S6M, Supporting Information). Additionally, in human intestinal tissues, no significant difference in MTCH2 expression was observed between colitis patients and healthy controls, according to the analysis of GEO datasets (GSE36807, GSE13367, and GSE48634) (Figure S6N, Supporting Information). These data raised the possibility that loss of MTCH2 might have no significant impact on colonic inflammation response. Taken together, these results demonstrated that MTCH2 deficiency inhibited intestinal tumorigenesis through TFRC‐mediated ferroptosis.

The populations and status of tumor and non‐tumor cell subsets play crucial roles in the regulation of tumor progression.^[^ ^34^ ^]^ To better understand the influence of MTCH2 deletion on the makeup of cell types and transcriptional patterns in CRC, we performed single‐cell RNA sequencing (scRNA‐seq) using CRC tissues harvested from MTCH2^cKO^ and MTCH2^fl/fl^ mice. Unsupervised t‐distributed stochastic neighbor embedding (tSNE) analysis using the Seurat R package identified 21 cell clusters based on gene expression patterns (Figure 7P; Figure S6O, Supporting Information). Next, using the CellMarker dataset and referencing previous studies,^[^ ^35^, ^36^ ^]^ we annotated these clusters into six cell types: B cells, cancer cells, endothelial cells, fibroblasts, myeloid cells, and T and natural killer (NK) cells (Figure 7Q; Figure S6P,Q, Supporting Information). Compared with the cell populations derived from wild‐type mice, we found increases in B cells, endothelial cells, myeloid cells, and T and NK cells, along with decreases in cancer cells and fibroblasts, in MTCH2^cKO^ mice (Figure 7R,S). These data suggested that tumor‐infiltrating immune cells may be involved in MTCH2‐mediated tumor formation.

Considering that the conditional knockout of MTCH2 applied to intestinal tissues, we focused on CRC tumor epithelial cells to describe the transcriptome landscape. Differentially expressed genes (DEGs) in cancer cells between MTCH2^cKO^ and MTCH2^fl/fl^ mice were extracted and visualized using a volcano plot (Figure 7T). As expected, the violin plot showed that MTCH2 expression was significantly downregulated in tumor cells of MTCH2^cKO^ mice, validating the knockout efficiency (Figure S6R, Supporting Information). Next, functional enrichment analyses were performed on the DEGs. Gene Ontology (GO) enrichment analysis revealed that the DEGs were enriched in several biological process terms, including negative regulation of cell population proliferation, cell morphogenesis, epithelial cell differentiation, small molecule biosynthetic process, and morphogenesis of an epithelium (Figure 7U). Additionally, Kyoto Encyclopedia of Genes and Genomes (KEGG) and Wiki pathway analyses revealed that the DEGs were involved in pathways related to antigen processing and presentation, focal adhesion, regulation of actin cytoskeleton, the oxidative stress and redox pathway, and signaling pathways regulating pluripotency of stem cells (Figure 7V). One of these, the oxidative stress and redox pathway, is a ferroptosis‐related biological pathway. Collectively, these results provided further evidence that MTCH2 depletion in tumor cells might enhance ferroptosis in CRC, leading to tumor suppression.

### 2.8 Depletion of MTCH2 Inhibits CRC Liver Metastasis and Enhances the Therapeutic Efficacy of Sorafenib

**Figure 8:**
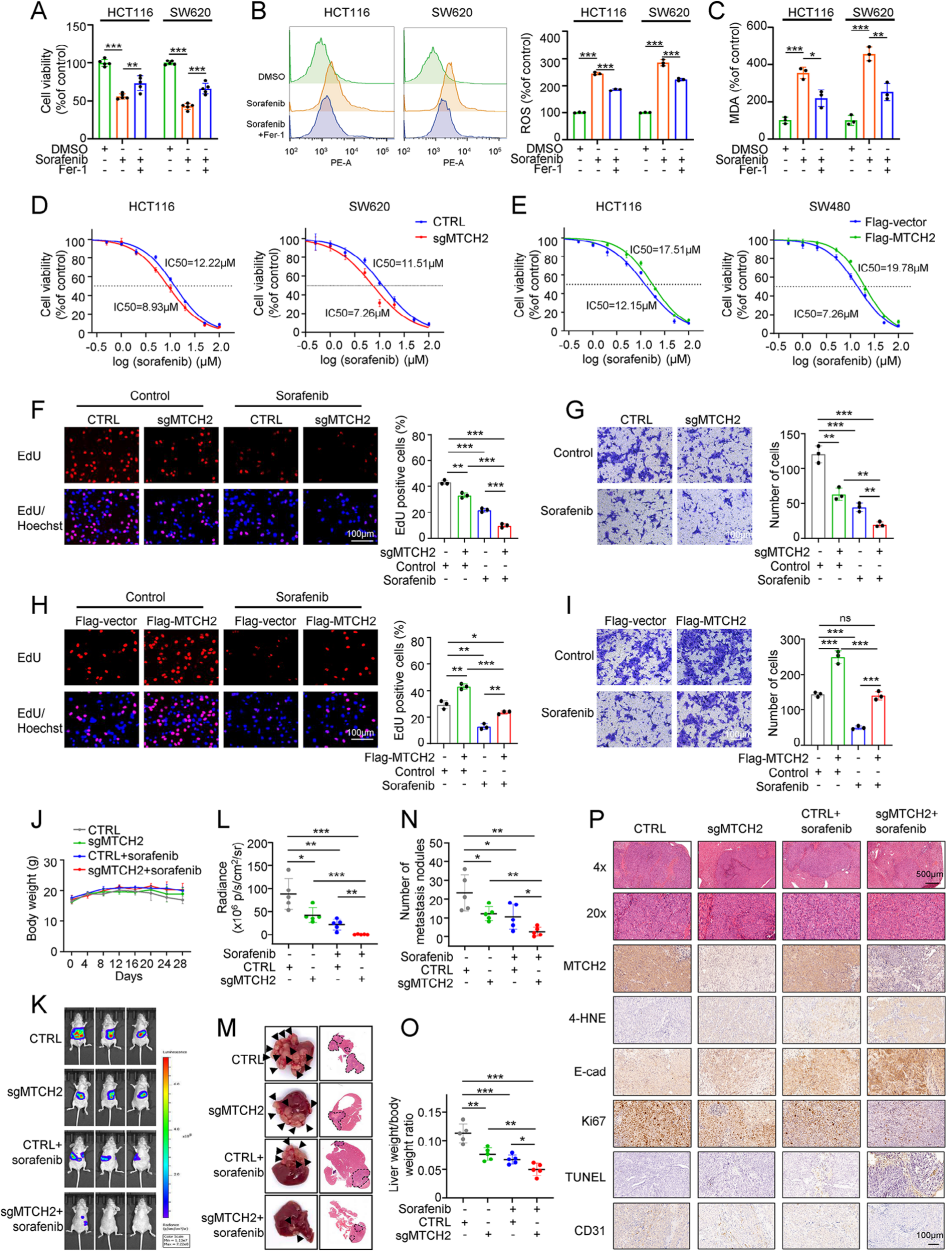
Loss of MTCH2 improves the response to sorafenib on the metastasis in vivo. A–C) Cell viability (A) and levels of ROS (B) and MDA (C) were measured in HCT116 and SW620 cells incubated with sorafenib (10 µm) and Fer‐1 (1 µm). D,E) CCK‐8 assay assessment of the half‐maximal inhibitory concentration (IC_50_) of sorafenib in MTCH2‐knockout (D) and MTCH2‐overexpressing (E) CRC cells. F,G) EdU (F) and Transwell (G) assays were performed in sgMTCH2 HCT116 cells incubated with sorafenib (10 µm). Scale bar, 100 µm. H,I) The proliferative (H) and migration (I) capacities were evaluated in MTCH2‐overexpressing HCT116 cells incubated with sorafenib (10 µm). Scale bar, 100 µm. J) The body weights of the four groups of mice with indicated treatment were recorded regularly (*n *= 5 in each group). K,L) Representative bioluminescence imaging of mice (K) with indicated treatment and quantification of the radiance intensity (L) at 4 weeks post‐implantation with HCT116 cells. M) Representative photographs of metastatic foci of mouse livers and images of H&E‐stained sections of metastatic lesions from each group. Arrows indicate liver metastasis foci. Dashed lines encircle metastatic foci on the liver surface. N,O) Quantification of metastatic liver nodules (N) and the ratio of liver weight to body weight (O). P) Representative images of H&E‐stained liver metastatic foci and IHC staining of MTCH2, 4‐HNE, Ki67, E‐cadherin (E‐cad), TUNEL, and CD31 in liver sections from each group. Scale bars, 500 and 100 µm. The statistical analysis was calculated by two‐way ANOVA for multiple comparisons and one‐way ANOVA with Tukey's honest difference post hoc test. Data present means ± SD from three independent experiments. *P < 0.05, **P < 0.01, ***P < 0.001; ns, not significant.

Accumulating evidence has demonstrated that sorafenib can induce ferroptosis, supporting its application as a therapeutic drug for liver metastasis and advanced liver cancer.^[^ ^37^, ^38^, ^39^ ^]^ However, whether sorafenib can induce ferroptosis in CRC cells is unclear. Thus, we first investigated the relationship between sorafenib and ferroptosis in CRC cells. Relative to no treatment, incubation with sorafenib reduced proliferation rates (**Figure** **8**A) and increased ROS and MDA levels (Figure 8B,C) in HCT116 and SW620 cells. Co‐treatment with Fer‐1 partially reversed these changes (Figure 8A–C). These results suggested that sorafenib can induce ferroptosis in CRC cells.

Considering the synergistic anti‐tumor effects of MTCH2 silencing and ferroptosis‐inducing agents in CRC cells, we next sought to investigate whether MTCH2 could affect sorafenib treatment of CRC liver metastasis. First, we found that MTCH2 knockout significantly decreased the half‐maximal inhibitory concentration (IC_50_) of sorafenib in HCT116 and SW620 cells (Figure 8D), whereas MTCH2 overexpression increased the IC_50_ dosage of sorafenib in HCT116 and SW480 cells (Figure 8E). EdU and Transwell assays proved that MTCH2 depletion combined with sorafenib remarkably suppressed tumor growth and migration (Figure 8F,G). Conversely, overexpression of MTCH2 led to the opposite results (Figure 8G,I). Similarly, compared with the MTCH2^fl/fl^ mouse tumor organoids, MTCH2^cKO^ organoids were found to be more sensitive to sorafenib (Figure S7A, Supporting Information).

To confirm these in vitro findings, a mouse model of liver metastasis was constructed by injecting the spleen with HCT116 cells, with or without MTCH2 knockout. Body weights were recorded regularly (Figure 8J). Two weeks after injection, bioluminescence imaging revealed the presence of liver metastasis (Figure S7B, Supporting Information). After three courses of treatment with sorafenib, bioluminescence imaging was carried out and mouse livers were collected for analysis. Similar to the in vitro results, MTCH2 silencing reduced the incidence of liver metastasis in both groups of mice (Figure 8K,L). Additionally, compared with the control tumor group, decreased numbers of liver metastatic nodules and lower liver weights were observed in the MTCH2 knockout tumor group (Figure 8M–O; Figure S7C, Supporting Information). Meanwhile, sorafenib treatment inhibited tumor metastasis compared with no treatment in both the control and MTCH2 knockout tumor groups (Figure 8K–O). Furthermore, MTCH2 knockout enhanced the inhibitory effect of sorafenib on CRC liver metastasis (Figure 8K–O). IHC analysis further demonstrated that silencing of MTCH2, either alone or in combination with sorafenib, increased the levels of 4‐HNE and E‐cadherin, and inhibited the expression of Ki67 (Figure 8P; Figure S7D, Supporting Information). Additionally, we found that sorafenib effectively increased apoptosis markers (TUNEL) and inhibited the angiogenesis markers (CD31) besides causing ferroptosis in CRC (Figure 8P; Figure S7D, Supporting Information). Collectively, these results suggested that the knockout of MTCH2 repressed the metastatic potential of CRC and enhanced the inhibitory effects of sorafenib on CRC liver metastasis in vivo.

## 3 Discussion

**Figure 9:**
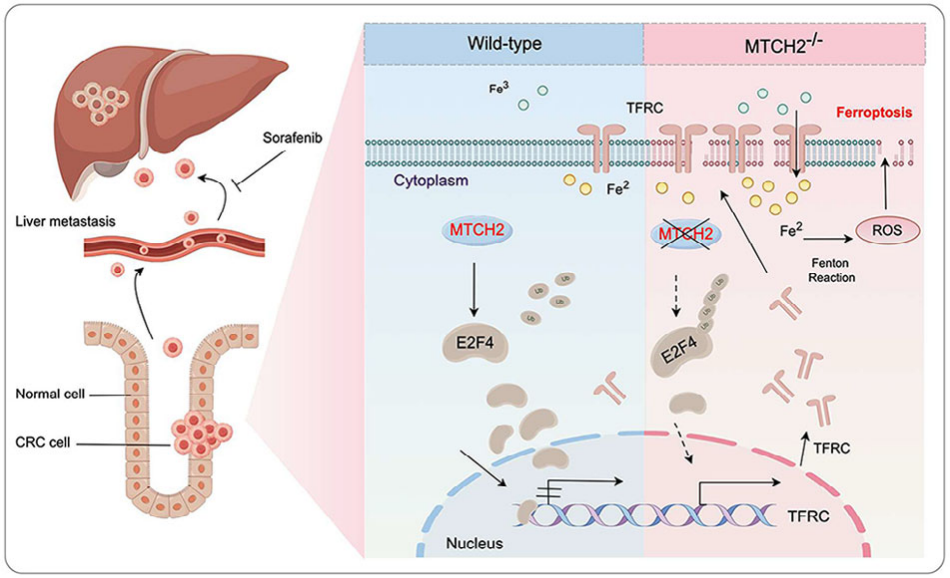
Schematic model of the potential mechanisms by which MTCH2 deficiency enhances ferroptosis and facilitates the effect of sorafenib on liver metastasis of CRC via the E2F4/TFRC axis. This graphical abstract was drawn using the Figdraw tool (www.figdraw.com).

Accumulating studies have demonstrated the crucial role of ferroptosis in the regulation of tumor occurrence and development,^[^ ^40^ ^]^ inspiring the search for novel ferroptosis‐related cancer treatment targets. In this study, we identified MTCH2 as a cancer‐promoting gene, with high MTCH2 levels predicting poor prognosis in patients with CRC. In cellular experiments and intestine‐specific MTCH2^cKO^ mice, the loss of MTCH2 induced ferroptosis via regulation of the E2F4‐TFRC axis. Additionally, MTCH2 knockout combined with sorafenib therapy showed potential as a therapeutic strategy for CRC liver metastasis (**Figure** **9**).

MTCH2 exhibits both inhibitory and promotive roles in different tumors, suggesting that it regulates distinct tumor‐related molecules or pathways.^[^ ^22^, ^23^, ^24^, ^25^ ^]^ In cervical carcinoma, loss of MTCH2 increased tumor cell proliferation by suppressing apoptosis.^[^ ^23^ ^]^ MiR‐135b/MTCH2 inhibition promoted the progression of erbB2‐driven mammary carcinomas.^[^ ^24^ ^]^ Conversely, silencing of MTCH2 decreased cell growth in ovarian cancer by downregulation of AIMP2 expression levels.^[^ ^41^ ^]^ In glioma cells, Yuan et al. found that MTCH2 knockdown impaired cell migration and invasion by repressing AKT signaling.^[^ ^25^ ^]^ Meanwhile, they observed a close correlation between MTCH2 expression and poor patient survival in glioma.^[^ ^25^ ^]^ Consistent with the study of gliomas, we found that higher MTCH2 expression exhibited poor outcomes.

Recent studies highlight MTCH2 as a critical regulator of mitochondrial metabolism and energy homeostasis in tumor progression. MTCH2 knockout inhibited the progression of non‐small cell lung cancer and ovarian cancer cells by impairing mitochondrial function, primarily reflected by lower ATP levels.^[^ ^41^, ^42^ ^]^ Inhibition of MTCH2 increased nuclear pyruvate and pyruvate dehydrogenase promoting the differentiation of AML cells.^[^ ^43^ ^]^ Moreover, the loss of MTCH2 in HeLa cells led to an increase in mitochondrial oxidative function) and enhanced fatty acid utilization.^[^ ^44^ ^]^ These findings suggest that MTCH2‐driven pathways and metabolic reprogramming are tissue‐specific, with distinct pathways dominating in different malignancies. In this study, we confirmed that depletion of MTCH2 markedly inhibited cell proliferation and metastasis of CRC in vivo and in vitro through regulation of iron metabolism and ferroptosis. Collectively, these data raised the possibility that MTCH2 is a critical promotional factor in CRC progression.

The close association between ferroptosis and tumor progression is well established. Therefore, the identification of ferroptosis‐related genes may provide new therapeutic targets. A recent study by Guo reported that MTCH2 suppresses ferroptosis by regulating mitochondrial permeability transition, highlighting its role in modulating pore dynamics at the organelle level.^[^ ^45^ ^]^ Our findings uncover a distinct transcriptional mechanism through which the MTCH2/E2F4/TFRC axis promotes CRC progression supported by in vivo evidence from intestine‐specific MTCH2^cKO^ mice. Moreover, our results support combining MTCH2 inhibition with sorafenib for CRC liver metastasis treatment. These data raise the possibility that MTCH2 may function as a potential target for the ferroptosis‐related treatments in CRC.

In addition to its role in tumor progression, increasing evidence demonstrates that ferroptosis is also implicated in diseases of other systems, including neurodegenerative diseases such as Alzheimer's and Parkinson's diseases.^[^ ^7^, ^46^ ^]^ Interestingly, previous studies have demonstrated that MTCH2 deficiency impairs the activity of neuronal cells and accelerates neurodegeneration,^[^ ^47^, ^48^ ^]^ together with our findings on the E2F4‐TFRC axis in MTCH2‐associated ferroptosis. All of these provide potential mechanistic insights into how MTCH2 may influence neurodegenerative disorders.

Among the many ferroptosis‐related molecules, we identified TFRC as one of the most relevant genes in MTCH2‐associated ferroptosis. Many transcription factors, including YAP, TP53, c‐MYC, GATA1, ETS1, and STAT5, reportedly participate in TFRC regulation.^[^ ^11^, ^49^, ^50^ ^]^ Here, we found that E2F4, as a transcription factor, participated in MTCH2‐mediated regulation of TFRC by inhibiting TFRC transcription and uncovered the potential E2F4‐binding site within the TFRC promoter.

Previous studies have shown that the E2F4 protein is unstable and can be degraded through the ubiquitin‐proteasome pathway.^[^ ^31^ ^]^ Therefore, we investigated the hypothesis that MTCH2 affects the ubiquitination of E2F4. Indeed, our evidence demonstrated that MTCH2 directly interacts with E2F4, resulting in deubiquitination and stabilization of the E2F4 protein. Therefore, we propose that MTCH2 knockout in CRC cells reduced E2F4 levels through the ubiquitin‐proteasome pathway, subsequently upregulating TFRC transcription levels and inducing ferroptosis.

Several studies assessed the effects of MTCH2 in model animals. MTCH2 knockout in zebrafish led to a decrease in the number of adipocytes.^[^ ^51^ ^]^ MTCH2 promoted lipid accumulation in transgenic MTCH2‐GFP mice by upregulating the levels of lipid metabolism genes such as FASN and SREBP1.^[^ ^18^ ^]^ Additionally, studies have shown that MTCH2 knockout in mice results in embryonic lethality.^[^ ^16^ ^]^ In this study, we evaluated for the first time the effects of MTCH2 on the progression of CRC in intestine‐specific MTCH2^cKO^ mice. Consistent with our in vitro findings, the loss of MTCH2 strongly reduced AOM/DSS‐induced tumorigenesis in these mice, demonstrating the oncogenic role of MTCH2 in CRC.

We also presented a comprehensive study of MTCH2‐mediated changes in the makeup of cell types and transcriptional patterns in CRC tissues of MTCH2^cKO^ mice. Our results revealed that the proportions of various cell types were significantly altered in MTCH2^cKO^ mice, providing a novel insight into the mechanisms underlying MTCH2‐regulated tumor progression. The pathway enrichment analysis of DEGs in tumor cells suggested a relationship between MTCH2 and the ferroptosis‐associated biological pathway, further supporting our in vitro results. Moreover, we observed that MTCH2 deficiency induced the alteration of multiple functions related to cell stemness and lipid homeostasis (Figure 7U,V), which is consistent with previous studies.^[^ ^20^, ^52^ ^]^

Metastasis is the primary fatality factor in patients with CRC.^[^ ^53^ ^]^ Several drugs that induce ferroptosis and inhibit tumor progression have been used clinically or entered into clinical trials.^[^ ^54^ ^]^ For example, cisplatin induces ferroptosis in lung cancer cells.^[^ ^55^ ^]^ Artemisinin was found to trigger ferroptosis in cancer cells^[^ ^56^ ^]^ and was well tolerated in patients with breast cancer and cervical intraepithelial neoplasia in several clinical trials (NCT00764036, NCT02354534).

As a multi‐kinase inhibitor, sorafenib induces ferroptosis of cancer cells.^[^ ^57^ ^]^ Multiple preclinical and clinical studies have demonstrated the activity of sorafenib against several types of tumors, including renal cell carcinoma, hepatocellular carcinoma (HCC), and CRC liver metastasis.^[^ ^58^, ^59^ ^]^ However, because of the heterogeneity of tumors, sorafenib exhibits variable efficacy for treating patients. Combining sorafenib with ferroptosis activators could promote the effect of sorafenib in cancer therapy. Several studies reported that enhancing ferroptosis through MT1G inhibitors or LCN2 antibodies increased the sensitivity of HCC to sorafenib.^[^ ^60^, ^61^ ^]^ Thus, identifying new targets that enhance the therapeutic effect of sorafenib may be an effective strategy for patients with tumors. Consistent with the synergistic inhibition of the growth of cervical cancer by targeting MTCH1 in combination with sorafenib treatment,^[^ ^62^ ^]^ we found that MTCH2 knockout synergistically facilitates the antitumor activity of sorafenib in CRC liver metastasis, indicating that the MTCH2 knockout and sorafenib had effects partially on distinct pathways.

This study has some limitations. Although our data revealed the clinical significance of MTCH2 in CRC patient samples, prospective studies that include cohorts with more detailed clinical information are needed to confirm our results. Additionally, due to the synergistic effect of MTCH2 depletion and sorafenib on liver metastasis, we speculate that MTCH2 could serve as a therapeutic indicator. Further studies are required to validate the potential of MTCH2‐targeted treatment for CRC patients with liver metastasis.

Overall, we provide important new insights into the pivotal role of MTCH2 in colorectal tumorigenesis and metastasis, specifically its mediation of ferroptosis via the E2F4‐TFRC axis. These findings suggest that MTCH2 may have potential as a novel therapeutic target for the regulation of ferroptosis in CRC patients.

## 4 Experimental Section

### Patients and Tissue Specimens

The human CRC tissue microarray containing a cohort of 172 patients was purchased from Shanghai Outdo Biotech. The other paraffin‐embedded tissue samples were obtained from the Department of Gastrointestinal Surgery IV, Peking University Cancer Hospital & Institute. This study was approved by the Research Ethics Committee of Peking University Cancer Hospital & Institute (2021KT136), and informed consent was obtained from all participants.

### Histological Analysis

Human CRC tissues, mouse subcutaneous tumors, and liver and intestinal tissues were fixed, paraffin‐embedded, sectioned at 4‐µm thickness, and stained with H&E. IHC staining was conducted following a previously reported protocol. Primary antibodies included anti‐MTCH2 (1:400 dilution, GTX130324; GeneTex, CA, USA), anti‐Ki67 (1:200, 12 202; Cell Signaling Technology, MA, USA), anti‐TFRC (1:2000, ab214039; Abcam, Cambridge, UK), anti‐E‐cad (1:400, 3195; Cell Signaling Technology, MA, USA), anti‐4‐HNE (1:200, bs‐6313R; Bioss, Beijing, China), anti‐GPX4 (1:100, ab125066; Abcam, Cambridge, UK), anti‐SLC7A11 (1:500, ab307601; Abcam, Cambridge, UK), and anti‐CD31 (1:4000, ab281583; Abcam, Cambridge, UK). IHC sections were photographed using NIS Elements software (Nikon, Tokyo, Japan). The immunohistochemical staining intensity was assessed by two independent, blinded expert pathologists. The staining intensity was scored as follows: 0 for no staining, 1 for weak staining, 2 for middle staining, and 3 for strong staining. The staining percentage positivity of positive cells was scored as follows: 0 for 0% stained, 1 for 1–25% stained, 2 for 26–50% stained, 3 for 51–75% stained, and 4 for 76–100% stained. IHC scores for MTCH2 were calculated by multiplying the percentage positivity of positive cells by the staining intensity. CRC patients were classified into low and high MTCH2 expression groups on the basis of these IHC scores.

### Measurement of Intracellular Fe^2+^

Intracellular Fe^2+^ in CRC cells or tissues was quantitatively assessed using the Fe^2+^ content assay kit (BC5415; Solarbio, Beijing, China), following the manufacturer's protocol. The FerroOrange probe (F374; Dojindo, Shanghai, China) was used to stain Fe^2+^ in live cells. Cells were initially incubated with 1 µM FerroOrange probe for 30 min at 37 °C, then incubated with Hoechst 33 342 dye for 15 min, and directly visualized under a confocal microscope.

### Determination of Cellular ROS, Lipid Peroxidation, and MDA Levels

Cellular ROS was detected by flow cytometry with a dihydroethidium (DHE) probe (50102ES25; Yeasen Biotech, Shanghai, China). Briefly, CRC cells were incubated in a culture medium with a 10 µm DHE probe at 37 °C for 30 min. Next, the cells were washed three times in phosphate‐buffered saline (PBS) and collected for fluorescence intensity detection. The generation of lipid peroxidation was evaluated by using BODIPY 581/591 C11 probe (S0043S; Beyotime, Shanghai, China) according to the manufacturer's instructions. MDA content was measured using the MDA assay kit (S0131M; Beyotime, Shanghai, China), in accordance with the manufacturer's protocol. Briefly, cells were resuspended in 200 µL lysis buffer and incubated on ice for 30 min. The lysate was centrifuged at 12 000 × *g* for 10 min at 4 °C. Next, 100 µL of the supernatant was incubated with 200 µL of MDA working solution at 100 °C for 15 min. After centrifugation at 1000 × *g* for 10 minutes at room temperature, 200 µL of the supernatant was added to a 96‐well plate for measurement of the absorbance at 532 nm using a microplate reader.

### Transmission Electron Microscopy

To examine mitochondrial morphology, CRC cells were fixed with 4% glutaraldehyde, embedded in epoxy resin, sectioned, and treated with lead citrate. TEM images were captured using a JEM‐1400 (JEOL, Tokyo, Japan) and analyzed by ImageJ software.

### Animal Experiments

All animal experiments were performed in accordance with the guidelines sanctioned by the Ethics Committee of the Peking University Cancer Hospital & Institute (Approval No. 2021KT134).

### Animal Study‐1

Cell‐derived xenograft experiments: Female BALB/c nude mice (age: 6 weeks) acquired from the Hua‐Fu‐Kang Corporation were used to investigate the effects of MTCH2 on tumor growth and metastasis in vivo. All mice were kept under specific pathogen‐free conditions.

### Animal Study‐2

To establish the subcutaneous xenograft model, 3 × 10^6^ RKO cells, with sgRNA control or sgMTCH2#1 knockout, were suspended in 100 µL PBS and subcutaneously injected into the flanks of the mice (*n* = 5 per group). Tumor growth was measured using calipers. Tumor volume was assessed with the following formula: volume = (length × width^2^)/2. After 25 days, the mice were euthanized by carbon dioxide inhalation to collect the tumor xenografts. The tumor tissues were fixed, embedded, and sectioned for H&E and IHC analyses.

### Animal Study‐3

To construct the liver metastasis model, 5 × 10^6^ HCT116 cells, with sgRNA control or sgMTCH2#1 knockout, were suspended in 50 µL PBS and subcutaneously injected into the spleens of the mice. Bioluminescence imaging was performed 14 days after cell injection using an IVIS (PerkinElmer). Once liver metastasis nodules were observed, the mice were randomized into four treatment groups (*n* = 5 per group): CTRL + saline, sgMTCH2 + saline, CTRL + sorafenib, and sgMTCH2 + sorafenib. In the sorafenib therapy groups, 10 mg kg^−1^ sorafenib tosylate (CSPC Ouyi Pharmaceutical Co., Ltd.), was administered by gavage every other day for 1 week. At 28 days post‐cell injection, liver metastasis was detected by live imaging using the IVIS system, and image radiance values were quantified with Living Image software (PerkinElmer). Subsequently, all mice were sacrificed, and the metastatic liver foci were counted. The liver tissues were then fixed and embedded for histological analysis.

### Animal Study‐4

Construction of conditional MTCH2 knockout mice and AOM/DSS modeling: MTCH2‐floxed C57BL/6 mice (MTCH2^fl/fl^) were created using a CRISPR/Cas9‐based genome‐editing system from Cyagen Biosciences. The sequence from exons 6 to 8 was selected as the target site. Subsequently, MTCH2^fl/fl^ mice were crossed with Villin‐Cre mice (Cyagen Biosciences, Guangzhou, China) to generate intestine‐conditional MTCH2 knockout (MTCH2^cKO^) mice. The genotype of MTCH2^cKO^ mice was confirmed by PCR amplification of tail genomic DNA. To generate the AOM/DSS‐induced CRC model, MTCH2^fl/fl^ and MTCH2^cKO^ mice (age: 6–8 weeks; half male and half female) were intraperitoneally administered with 10 mg kg^−1^ AOM (A5486; Sigma‐Aldrich, MO, USA) and maintained with regular drinking water for 1 week. The mice were then subjected to DSS (021 601 1080; MP Biomedicals, CA, USA) as follows: three cycles of 2.5% DSS‐treated water for 1 week and regular water for 2 weeks. Body weight and occult blood were recorded every 3 days. Twelve weeks after AOM administration, the animals were euthanized by carbon dioxide inhalation to collect the colorectum. The length of the colon and the number of tumors were measured, and the tumor tissues were fixed and embedded for histological analysis.

### Tissue Processing and scRNA‐Seq

Fresh tumor samples obtained from groups of MTCH2^fl/fl^ and MTCH2^cKO^ mice were pooled, rinsed with PBS, minced, and dissociated with 1× Trypsin‐EDTA solution for 20 min at 37 °C. A Countstar Fluorescence Cell Analyzer was used to assess the cell viability of dissociated single cells. The cDNA synthesis and library construction for scRNA‐seq strictly adhered to the protocols of the respective manufacturers. Briefly, unique RNAs released from individual cells underwent barcoding by reverse transcription in individual gel bead‐in emulsions. This process yielded cDNA, which was then amplified and quality‐checked to ensure accuracy. Subsequently, the libraries were sequenced on an Illumina Novaseq 6000 platform, employing a paired‐end 150‐bp strategy that achieved a minimum sequencing depth of 100 000 reads per cell to guarantee comprehensive coverage and analysis by CapitalBio Technology.

### Processing of scRNA‐Seq Data

Cell Ranger software was employed for comprehensive data processing, encompassing alignment, filtering, barcode enumeration, and unique molecular identifier counting via the count module. This streamlined process generated a feature‐barcode matrix and identified clusters, which were then converted into Seurat objects using the Seurat package (v3.0). Cells exhibiting a low gene count (<200), high gene expression rank (top 1% percentile), or excessive mitochondrial gene contributions (>25%) were flagged as abnormal and excluded from further analysis. Dimensionality reduction was applied through principal component analysis, while data visualization was accomplished by tSNE. Expression of the following marker genes was used to score cell clusters: B cells (Cd79a, Cd79b, Cd19, Cd22, Ms4a1), cancer cells (Epcam, Cdh1, Krt8, Krt18), endothelial cells (Cdh5, Ramp2, Eng), fibroblasts (Col1a2, Col1a1, Dcn), myeloid cells (Apoe, C1qc, S100A8, S100a9, Csf3r), and T and NK cells (Cd3e, Cd3d, Cd3g, Trbc2, Icos). GO and pathway enrichment analyses of DEGs in tumor cells from MTCH2^fl/fl^ and MTCH2^cKO^ mice were performed using the Metascape tool (https://metascape.org/gp/index.html) and visualized using the R package.

### Statistical Analysis

All statistical analyses were conducted using GraphPad Prism software (version 8.0) or SPSS software (version 23.0). Survival analysis was performed using a Kaplan–Meier curve and a log‐rank test. Pearson's correlation test was used to evaluate the association between MTCH2 and TFRC. Two groups were compared by two‐tailed Student's *t*‐tests, while more than two groups were assessed by one‐way ANOVA with Tukey's honest difference post hoc test or two‐way ANOVA. Data were presented as means ± SD, and all experiments were conducted at least three times. A *P‐*value < 0.05 was defined as the threshold for statistical significance.

## Conflict of Interest

The authors declare no conflict of interest.

## Author Contributions

P.X., B.J., and X.S. conceived and designed the study. P.X. conducted most of the analysis, experiments and revision of the manuscript. J.C., H.H., X.Q., X.Y., K.W., J.C., L.S., T.L., Y.H., T.S., Y.R., B.C., H.Y., W. Z., Z.W., and J.D. participated experimental study and reviewed the manuscript; J.C., H.Y., W. Z., B.J., and X.S. funded the study; B.J. and X.S. supervised and reviewed the manuscript. All authors read and approved the final manuscript.

## Supporting information



Supporting Information

Supporting Information

Supporting Information

Supporting Information

Supporting Information

Supporting Information

Supporting Information

Supporting Information

Supporting Information

## Data Availability

The data that support the findings of this study are available from the corresponding author upon reasonable request.

## References

[1] H. Sung , J. Ferlay , R. L. Siegel , M. Laversanne , I. Soerjomataram , A. Jemal , F. Bray , CA Cancer J. Clin. 2021, 71, 209. 33538338 10.3322/caac.21660

[2] C. Carlomagno , A. De Stefano , M. Rosanova , S. De Falco , L. Attademo , G. Fiore , S. De Placido , Cancer Metastasis Rev. 2019, 38, 307. 30003458 10.1007/s10555-018-9748-7

[3] D. C. Osei‐Bordom , S. Kamarajah , N. Christou , Biomedicines 2021, 9, 894. 34440099 10.3390/biomedicines9080894 PMC8389538

[4] S. J. Dixon , K. M. Lemberg , M. R. Lamprecht , R. Skouta , E. M. Zaitsev , C. E. Gleason , D. N. Patel , A. J. Bauer , A. M. Cantley , W. S. Yang , B. Morrison , B. R. Stockwell , Cell 2012, 149, 1060. 22632970 10.1016/j.cell.2012.03.042 PMC3367386

[5] Q. Yu , N. Zhang , X. Gan , L. Chen , R. Wang , R. Liang , J. Jian , Phytomedicine 2023, 119, 154999. 37597361 10.1016/j.phymed.2023.154999

[6] C. Zhang , X. Liu , S. Jin , Y. Chen , R. Guo , Mol. Cancer 2022, 21, 47. 35151318 10.1186/s12943-022-01530-y PMC8840702

[7] S. K. Ryan , C. L. Ugalde , A. S. Rolland , J. Skidmore , D. Devos , T. R. Hammond , Trends Pharmacol. Sci. 2023, 44, 674. 37657967 10.1016/j.tips.2023.07.007

[8] E. Thomas , E. Roman , S. Claypool , N. Manzoor , J. Pla , S. L. Panwar , Antimicrob. Agents Chemother. 2013, 57, 5580. 23979757 10.1128/AAC.00889-13 PMC3811284

[9] J. Wang , K. Pantopoulos , Biochem. J. 2011, 434, 365. 21348856 10.1042/BJ20101825 PMC3048577

[10] G. Xing , L. Meng , S. Cao , S. Liu , J. Wu , Q. Li , W. Huang , L. Zhang , EMBO Rep. 2022, 23, 52280. 10.15252/embr.202052280 PMC9346473 35703725

[11] J. Wu , A. M. Minikes , M. Gao , H. Bian , Y. Li , B. R. Stockwell , Z. N. Chen , X. Jiang , Nature 2019, 572, 402. 31341276 10.1038/s41586-019-1426-6 PMC6697195

[12] H. Feng , K. Schorpp , J. Jin , C. E. Yozwiak , B. G. Hoffstrom , A. M. Decker , P. Rajbhandari , M. E. Stokes , H. G. Bender , J. M. Csuka , P. S. Upadhyayula , P. Canoll , K. Uchida , R. K. Soni , K. Hadian , B. R. Stockwell , Cell Rep. 2020, 30, 3411. 32160546 10.1016/j.celrep.2020.02.049 PMC7172030

[13] L. Yi , Y. Hu , Z. Wu , Y. Li , M. Kong , Z. Kang , B. Zuoyuan , Z. Yang , Cell Death Dis. 2022, 13, 592. 35821227 10.1038/s41419-022-05027-w PMC9276735

[14] M. Hiromatsu , K. Toshida , S. Itoh , N. Harada , K. Kohashi , Y. Oda , T. Yoshizumi , Ann. Surg. Oncol. 2023, 30, 8675. 37548836 10.1245/s10434-023-14053-7

[15] K. Labbé , S. Mookerjee , M. L. Vasseur , E. Gibbs , C. Lerner , J. Nunnari , J. Cell Biol. 2021, 220, 202103122. 10.1083/jcb.202103122 PMC8496048 34586346

[16] Y. Zaltsman , L. Shachnai , N. Yivgi‐Ohana , M. Schwarz , M. Maryanovich , R. H. Houtkooper , F. M. Vaz , F. De Leonardis , G. Fiermonte , F. Palmieri , B. Gillissen , P. T. Daniel , E. Jimenez , S. Walsh , C. M. Koehler , S. S. Roy , L. Walter , G. Hajnóczky , A. Gross , Nat. Cell Biol. 2010, 12, 553. 20436477 10.1038/ncb2057 PMC4070879

[17] M. Maryanovich , Y. Zaltsman , A. Ruggiero , A. Goldman , L. Shachnai , S. L. Zaidman , Z. Porat , K. Golan , T. Lapidot , A. Gross , Nat. Commun. 2015, 6, 7901. 26219591 10.1038/ncomms8901

[18] Y. Bar‐Lev , S. Moshitch‐Moshkovitz , G. Tsarfaty , D. Kaufman , J. Horev , J. H. Resau , I. Tsarfaty , PLoS One 2016, 11, 0157850. 10.1371/journal.pone.0157850 PMC4928869 27359329

[19] A. Ruggiero , E. Aloni , E. Korkotian , Y. Zaltsman , E. Oni‐Biton , Y. Kuperman , M. Tsoory , L. Shachnai , S. Levin‐Zaidman , O. Brenner , M. Segal , A. Gross , Sci. Rep. 2017, 7, 44401. 28276496 10.1038/srep44401 PMC5343590

[20] A. Bahat , A. Goldman , Y. Zaltsman , D. H. Khan , C. Halperin , E. Amzallag , V. Krupalnik , M. Mullokandov , A. Silberman , A. Erez , A. D. Schimmer , J. H. Hanna , A. Gross , Nat. Commun. 2018, 9, 5132. 30510213 10.1038/s41467-018-07519-w PMC6277412

[21] A. Guna , T. A. Stevens , A. J. Inglis , J. M. Replogle , T. K. Esantsi , G. Muthukumar , K. C. L. Shaffer , M. L. Wang , A. N. Pogson , J. J. Jones , B. Lomenick , T. F. Chou , J. S. Weissman , R. M. Voorhees , Science 2022, 378, 317. 36264797 10.1126/science.add1856 PMC9674023

[22] X. Peng , Y. Yang , R. Hou , L. Zhang , C. Shen , X. Yang , Z. Luo , Z. Yin , Y. Cao , Drug Des. Dev. Ther. 2024, 18, 2203. 10.2147/DDDT.S460448 PMC11180440 38882047

[23] T. Zhang , Y. Hu , T. Wang , P. Cai , Int. J Mol. Med. 2017, 40, 21. 28498397 10.3892/ijmm.2017.2980 PMC5466377

[24] M. Arigoni , G. Barutello , F. Riccardo , E. Ercole , D. Cantarella , F. Orso , L. Conti , S. Lanzardo , D. Taverna , I. Merighi , R. A. Calogero , F. Cavallo , E. Quaglino , Am. J Pathol. 2013, 182, 2058. 23623609 10.1016/j.ajpath.2013.02.046

[25] Q. Yuan , W. Yang , S. Zhang , T. Li , M. Zuo , X. Zhou , J. Li , M. Li , X. Xia , M. Chen , Y. Liu , Mol. Med. 2021, 27, 7. 33509092 10.1186/s10020-020-00261-4 PMC7842075

[26] R. Li , H. He , X. He , Exp. Ther. Med. 2023, 25, 163. 36911382 10.3892/etm.2023.11862 PMC9996334

[27] L. Wang , Y. Liu , T. Du , H. Yang , L. Lei , M. Guo , H. F. Ding , J. Zhang , H. Wang , X. Chen , C. Yan , Cell Death Differ. 2020, 27, 662. 31273299 10.1038/s41418-019-0380-z PMC7206049

[28] X. Jiang , B. R. Stockwell , M. Conrad , Nat. Rev. Mol. Cell Biol. 2021, 22, 266. 33495651 10.1038/s41580-020-00324-8 PMC8142022

[29] Y. Lu , Q. Yang , Y. Su , Y. Ji , G. Li , X. Yang , L. Xu , Z. Lu , J. Dong , Y. Wu , J. X. Bei , C. Pan , X. Gu , B. Li , Cell Death Dis. 2021, 12, 511. 34011924 10.1038/s41419-021-03790-w PMC8134466

[30] T. M. Djajawi , L. Liu , J. N. Gong , A. S. Huang , M. J. Luo , Z. Xu , T. Okamoto , M. J. Call , D. C. S. Huang , M. F. van Delft , Cell Death Differ. 2020, 27, 2484. 32094511 10.1038/s41418-020-0517-0 PMC7370232

[31] W. Xiao , J. Wang , X. Wang , S. Cai , Y. Guo , L. Ye , D. Li , A. Hu , S. Jin , B. Yuan , Y. Zhou , Q. Li , Q. Tong , L. Zheng , Autophagy 2022, 18, 2615. 35253629 10.1080/15548627.2022.2044651 PMC9629121

[32] J. Hsu , J. Sage , Cell Cycle 2016, 15, 3183. 27753528 10.1080/15384101.2016.1234551 PMC5176148

[33] J. Liu , L. Xia , S. Wang , X. Cai , X. Wu , C. Zou , B. Shan , M. Luo , D. Wang , J. Cancer 2021, 12, 5173. 34335934 10.7150/jca.53708 PMC8317516

[34] F. Wu , J. Fan , Y. He , A. Xiong , J. Yu , Y. Li , Y. Zhang , W. Zhao , F. Zhou , W. Li , J. Zhang , X. Zhang , M. Qiao , G. Gao , S. Chen , X. Chen , X. Li , L. Hou , C. Wu , C. Su , C. Zhou , Nat. Commun. 2021, 12, 2540. 33953163 10.1038/s41467-021-22801-0 PMC8100173

[35] X. Zhang , Y. Lan , J. Xu , F. Quan , E. Zhao , C. Deng , T. Luo , L. Xu , G. Liao , M. Yan , Y. Ping , F. Li , A. Shi , J. Bai , T. Zhao , X. Li , Y. Xiao , Nucleic Acids. Res. 2019, 47, D721. 30289549 10.1093/nar/gky900 PMC6323899

[36] S. Chen , L. Fan , Y. Lin , Y. Qi , C. Xu , Q. Ge , Y. Zhang , Q. Wang , D. Jia , L. Wang , J. Si , L. Wang , Cancer Commun. 2023, 43, 1027. 10.1002/cac2.12469 PMC10508156 37533188

[37] X. Xu , Y. Li , Y. Wu , M. Wang , Y. Lu , Z. Fang , H. Wang , Y. Li , Redox Biol. 2023, 59, 102564. 36473315 10.1016/j.redox.2022.102564 PMC9723522

[38] M. Mu , C. X. Huang , C. Qu , P. L. Li , X. N. Wu , W. Yao , C. Shen , R. Huang , C. C. Wan , Z. W. Jian , L. Zheng , R. Q. Wu , X. M. Lao , D. M. Kuang , Cancer Res. 2024, 84, 841. 38231484 10.1158/0008-5472.CAN-23-1796

[39] Y. J. Chang , W. H. Hsu , C. H. Chang , K. L. Lan , G. Ting , T. W. Lee , Mol. Clin. Oncol. 2014, 2, 380. 24772304 10.3892/mco.2014.246 PMC3999117

[40] G. Lei , L. Zhuang , B. Gan , Nat. Rev. Cancer 2022, 22, 381. 35338310 10.1038/s41568-022-00459-0 PMC10243716

[41] G. Sun , Y. Song , C. Li , B. Sun , C. Li , J. Sun , P. Xiao , Z. Zhang , Oncol. Lett. 2024, 28, 492. 39185493 10.3892/ol.2024.14625 PMC11342418

[42] Y. Zhao , S. Wu , G. Cao , P. Song , C. G. Lan , L. Zhang , Y. H. Sang , Cell Death Dis. 2025, 16, 95. 39948081 10.1038/s41419-025-07419-0 PMC11825924

[43] D. H. Khan , M. Mullokandov , Y. Wu , V. Voisin , M. Gronda , R. Hurren , X. Wang , N. MacLean , D. V. Jeyaraju , Y. Jitkova , G. W. Xu , R. Laister , A. Seneviratne , Z. M. Blatman , T. Ketela , G. D. Bader , S. A. Marhon , D. D. De Carvalho , M. D. Minden , A. Gross , A. D. Schimmer , Blood 136, 81. 32299104 10.1182/blood.2019000106

[44] S. Chourasia , C. Petucci , C. Shoffler , D. Abbasian , H. Wang , X. Han , E. Sivan , A. Brandis , T. Mehlman , S. Malitsky , M. Itkin , A. Sharp , R. Rotkopf , B. Dassa , L. Regev , Y. Zaltsman , A. Gross , EMBO J. 2025, 44, 1007. 39753955 10.1038/s44318-024-00335-7 PMC11832942

[45] L. Guo , FEBS Lett. 2025, 599, 352. 39227319 10.1002/1873-3468.15008

[46] M. Jakaria , A. A. Belaidi , A. I. Bush , S. Ayton , J. Neurochem. 2021, 159, 804. 34553778 10.1111/jnc.15519

[47] J. Gockley , K. S. Montgomery , W. L. Poehlman , J. C. Wiley , Y. Liu , E. Gerasimov , A. K. Greenwood , S. K. Sieberts , A. P. Wingo , T. S. Wingo , L. M. Mangravite , B. A. Logsdon , Genome Med. 2021, 13, 76. 33947463 10.1186/s13073-021-00890-2 PMC8094491

[48] M. C. Stephens , J. Li , M. Mair , J. Moore , K. Zhu , A. . Tarkunde , B. Amoh , A. M. Perez , A. Bhakare , F. Guo , J. M. Shulman , I. Al‐Ramahi , J. Botas , Am. J. Hum. Genet. 2025, 112, 1081. 40215969 10.1016/j.ajhg.2025.03.012 PMC12120185

[49] J. P. Holland , M. J. Evans , S. L. Rice , J. Wongvipat , C. L. Sawyers , J. S. Lewis , Nat. Med. 2012, 18, 1586. 23001181 10.1038/nm.2935 PMC3521603

[50] L. J. Tang , Y. J. Zhou , X. M. Xiong , N. S. Li , J. J. Zhang , X. J. Luo , J. Peng , Biol. Med. 2021, 162, 339. 10.1016/j.freeradbiomed.2020.10.307 33157209

[51] K. Landgraf , A. Strobach , W. Kiess , A. Körner , FEBS Lett. 2016, 590, 2852. 27468124 10.1002/1873-3468.12330

[52] V. Rottiers , A. Francisco , M. Platov , Y. Zaltsman , A. Ruggiero , S. S. Lee , A. Gross , S. Libert , Obesity 2017, 25, 616. 28127879 10.1002/oby.21751

[53] R. S. Zheng , R. Chen , B. F. Han , S. M. Wang , L. Li , K. X. Sun , H. M. Zeng , W. W. Wei , J. He , Chin. J. Oncol. 2024, 46, 221. 10.3760/cma.j.cn112152-20240119-00035 38468501

[54] X. Chen , R. Kang , G. Kroemer , D. Tang , Nat. Rev. Clin. Oncol. 2021, 18, 280. 33514910 10.1038/s41571-020-00462-0

[55] C. Gao , F. Xiao , L. Zhang , Y. Sun , L. Wang , X. Liu , H. Sun , Z. Xie , Y. Liang , Q. Xu , L. Wang , Ann. Transl. Med. 2022, 10, 224. 35280420 10.21037/atm-21-6909 PMC8908163

[56] R. Lin , Z. Zhang , L. Chen , Y. Zhou , P. Zou , C. Feng , L. Wang , G. Liang , Cancer Lett. 2016, 381, 165. 27477901 10.1016/j.canlet.2016.07.033

[57] L. Zhang , X. M. Li , X. H. Shi , K. Ye , X. L. Fu , X. Wang , S. M. Guo , Q. Ma , J. F. F. Xu , H. M. Sun , Q. Q. Li , W. Y. Zhang , L. H. Ye , Acta Pharmacol. Sin. 2023, 44, 622. 36109580 10.1038/s41401-022-00981-9 PMC9958095

[58] M. N. Fishman , J. Tomshine , W. J. Fulp , P. K. Foreman , PLoS One 2015, 10, 0120877. 10.1371/journal.pone.0120877 PMC4382117 25830512

[59] R. Gao , R. K. R. Kalathur , M. Coto‐Llerena , C. Ercan , D. Buechel , S. Shuang , S. Piscuoglio , M. T. Dill , F. D. Camargo , G. Christofori , F. Tang , EMBO Mol. Med. 2021, 13, 14351. 10.15252/emmm.202114351 PMC8649869 34664408

[60] X. Sun , X. Niu , R. Chen , W. He , D. Chen , R. Kang , D. Tang , Hepatology 2016, 64, 488. 27015352 10.1002/hep.28574 PMC4956496

[61] F. Yao , Y. Deng , Y. Zhao , Y. Mei , Y. Zhang , X. Liu , C. Martinez , X. Su , R. R. Rosato , H. Teng , Q. Hang , S. Yap , D. Chen , Y. Wang , M. M. Chen , M. Zhang , H. Liang , D. Xie , X. Chen , H. Zhu , L. Ma , Nat. Commun. 2021, 12, 7333. 34921145 10.1038/s41467-021-27452-9 PMC8683481

[62] X. Wang , Y. Ji , J. Qi , S. Zhou , S. Wan , C. Fan , Z. Gu , P. An , Y. Luo , J. Luo , Cell Death Dis. 2023, 14, 508. 37550282 10.1038/s41419-023-06033-2 PMC10406804

