# Supplementary material for: MTCH2 Deficiency Promotes E2F4/TFRC‐Mediated Ferroptosis and Sensitizes Colorectal Cancer Liver Metastasis to Sorafenib
Source: Adv Sci (Weinh). 2025 Jul 2;12(36):e00019. doi: 10.1002/advs.202500019 (PMC12463053; doi:10.1002/advs.202500019)
Supplement: Supplementary file 1 — Supporting Information [file ADVS-12-e00019-s005.docx]

**MTCH2 Deficiency Promotes E2F4/TFRC-Mediated Ferroptosis and Sensitizes Colorectal Cancer Liver Metastasis to Sorafenib**

*Pu Xing^1^, Jiangbo Chen^1^, Hao Hao^1^, Xiaowen Qiao^1^, Xinying Yang^1^, Kai Weng^1^, Jie Chen^2^,*

*Lin Song^1^, Tianqi Liu^1,3^, Yifan Hou^1^, Tongkun Song^1^, Yumeng Ran^1^, Bo Chen^1^, Hong Yang^1,4^, Wei Zhao^5^, Zaozao Wang^1^, Jiabo Di^1^, Beihai Jiang^1,*^, Xiangqian Su^1,6,*^*

*Corresponding authors.

**Supplementary methods**

**Quantitative real‐time PCR (qRT‐PCR):** Total RNA, extracted from CRC tissues or cells using Trizol (Invitrogen, Karlsruhe, Germany), was reversely transcribed into cDNA by a reverse transcription kit (Promega, WI, USA). qRT-PCR was conducted following previously described protocol,^[1]^ and normalized with GAPDH. All primers employed in this study are shown in Table S4.

**Western blot and cell immunofluorescence staining:** Protein extraction and western blot were carried out as described previously.^[2]^ Among them, nuclear and cytosolic proteins were isolated with the Nuclear and Cytoplasmic Protein Extraction Kit (P0028; Beyotime, Shanghai, China) based on the product instruction. For cell immunofluorescence staining, cells were seeded with sterile glass coverslips in 6-well plates and incubated for 36 hours. Next, cells were then fixed with 4% paraformaldehyde, permeabilized in 0.5% Triton X-100, and blocked in 5% BSA at room temperature, followed by overnight incubation with primary antibodies at 4°C. Secondary antibodies were incubated at room temperature. 647-conjugated TOM20 Rabbit mAb (A26784; Abclonal, Wuhan, China) was used for fluorescent labeling of mitochondria. The cell nuclei were stained with DAPI, and images were captured using an LSM780 confocal microscope (Zeiss, Oberkochen, Germany). The primary antibodies used in this study are shown in Table S5.

**Cell culture and reagents:** The human intestinal epithelial cell line (NCM460) and CRC cell lines (RKO, HCT116, LoVo, SW480, and SW620) were obtained from American Type Culture Collection (ATCC). Cells were cultured in RPMI or DMEM high glucose medium (HyClone, UT, USA) enriched with 10% FBS and 1% Penicillin/Streptomycin in a humidified incubator maintained at 37°C with 5% CO_2_. Fer-1 (S7243; Selleck Chemical), Z-VAD-FMK (S7023; Selleck Chemical, Shanghai, China), Nec-1 (S8037; Selleck Chemical), Erastin (S7242; Selleck Chemical), E2F4i (S8963; Selleck Chemical), and Sorafenib (S7397; Selleck Chemical) were obtained from Selleck Chemical. Cycloheximide (HY-12320; MedChemExpress, NJ, USA) and MG-132 (HY-13259; MedChemExpress) were purchased from MedChemExpress.

**Plasmid, siRNA, and lentivirus transfection:** For overexpression, the MTCH2 coding sequence was cloned into the pCMV-3Tag-1A plasmid. The cDNA of the full-length E2F4 sequence was inserted into the pCMV-Myc vector. For knockdown, the TFRC-siRNA#1 (5’- GTAGGATGGTAACCTCAGA -3’) and TFRC-siRNA#2 (5’- GGAGACTTCTTCCGTGCTA -3’) were purchased from Ribobio Biological. For knockout of MTCH2, two single-guide RNA (sgRNA) targeting MTCH2 (sgMTCH2#1: 5’- TTCGGGAGTCCTTGGAACTG -3’; sgMTCH2#2: 5’- CATGAGCGGCTGGGACAGGA -3′) were acquired from Shanghai Jikai Gene Chemical Technology. SMARTpool MTCH2 siRNA purchased from Dharmacon (Cat #L-007371-00-0005), which contains a mixture of four independent siRNAs targeting the same gene, with proprietary chemical modifications to minimize off-target effects. For plasmid and siRNA transfection, cells were transfected with Lipofectamine 2000 (Invitrogen, California, USA) according to the manufacturer’s suggestions. As for sgMTCH2 lentiviral transfection, cells were infected for 72 hours and then selected using 2 μg/mL puromycin for 7 days.

**Cell proliferation, migration, and invasion assay:** Cell proliferation ability was assessed by CCK8, clone formation assay, and EdU staining. CCK8 and clone formation assays were carried out as previously described.^[3]^ EdU staining was carried out by an EdU kit conjugated with Alexa Fluor 594 (C0078L; Beyotime Biotechnology, Shanghai, China). Briefly, CRC cells were seeded in 24-well plates at a density of 5 × 10^4^ cells per well and subsequently treated with 10 μM EdU for 2 hours at 37°C. Following this, cells were fixed in 4% paraformaldehyde and permeabilized using 0.5% TritonX-100 for 20 minutes. Next, the click reaction was performed in a dark environment. Finally, the cells were stained with Hoechst 33342 dye for 15-30 minutes and observed under a fluorescence microscope. Cell migration and invasion ability were determined by 24-well transwell chambers (Corning, NY, USA). The cells were suspended in serum-free medium and planted in transwell chambers with or without matrigel. And add complete culture medium in the lower chamber. Following 24 hours of incubation, migrated cells were fixed with 4% paraformaldehyde, stained with crystal violet, and counted with an invert microscope.

**Co-immunoprecipitation (CO-IP) and ubiquitination Assays:** The Co-IP assay was performed as described previously.^[4]^ As for ubiquitination analysis, HEK293T cells were transfected with ubiquitin and substrate plasmid, including Myc-E2F4, Flag-MTCH2 or Flag-vector. After 48 hours, the cells were incubated with MG132 (10μM) for 8 hours. Subsequently, cells were lysed with lysis buffer, and immunoprecipitated with c-Myc antibody. The ubiquitination condition of the E2F4 protein was assessed by western blot using the anti-ubiquitin antibody.

**Mitochondrial fractionation:** Mitochondrial and cytosolic fractions were isolated from CRC cells using the Cell Mitochondria Isolation Kit (C3601; Beyotime Biotechnology, Shanghai, China) according to the manufacturer’s protocols. In brief, cells were harvested and resuspended in mitochondrial isolation buffer for 15 min, then subjected to Dounce homogenization. The cells were homogenized by 30 strokes and centrifuged at 600 × g for 10 min at 4 °C to obtain mitochondrial suspension. The supernatant was centrifuged at 11,000 × g for 10 min at 4 °C to obtain mitochondria. For protein cross-linking experiments, we performed formaldehyde cross-linking of intact cells. Subsequently, the mitochondrial protein was extracted in the absence of detergents.

**Chromatin immunoprecipitation (ChIP) assays:** ChIP assay was performed as described previously through the SimpleChIP® Plus Sonication Chromatin IP Kit (9002; CST, MA, USA). E2F4 antibody (10923-1-AP; Proteintech, IL, USA) was used for ChIP. All primers employed in this study are shown in Table S4.

**Luciferase reporter assay:** The JASPAR database was used to predict potential binding sites of E2F4 in the TFRC promoter within the 1000bp proximal to the transcription start site of TFRC. The TFRC promoter fragments from −1000bp to 0bp, along with deletion mutants harboring deletions of the corresponding E2F4-binding sites (mut1: −746bp to −732bp, and mut2: −203bp to −190bp), were cloned into the pGL3-basic plasmid. Cells were seeded into 24-well plates and subsequently transfected with luciferase reporter vectors. After 48 hours of transfection, the cells were harvested, and luciferase activity was determined with the Dual-Luciferase Reporter Assay System (Promega, WI, USA), following the manufacturer's guidelines.

**Mouse-derived intestinal cancer organoid:** Tumor tissues from mouse colon were dissected into small fragments and washed with PBS buffer. Following tissue dissociation in EDTA solution, the suspension was centrifuged at 400 × g for 5 min, and the resulting single cells were resuspended in Matrigel (Corning, 356255). 20 μl of Matrigel-cell mixture was plated per well in a prewarmed 48-well plate. After polymerization, each well was supplemented with 300 μl of mouse intestinal organoid culture medium (Mogengel, MA-0807T001LP). Organoids were allowed to grow until formation, at which point uniformly sized structures were selected for further analysis. Medium changes were performed every 2-3 days. Organoid dimensions were quantified using ImageJ. For histological evaluation, the organoids were fixed in 4% paraformaldehyde for 30 min, paraffin-embedded, and sectioned at 4 μm thickness for subsequent staining.

**DSS-induced inflammation cell model:** NCM460 cells were plated in six-well plates until cell confluence attained 70% and exposed to 2% DSS for 24 h. Subsequently, the cells were collected and their RNA was extracted for qRT-PCR as described above. Inflammatory response indicators included IL-1β and IL-6.

**TUNEL assay:** Apoptosis was detected using TUNEL Apoptosis Detection kit (C1091; Beyotime Biotechnology, Shanghai, China) following the manufacturer’s protocols. Briefly, paraffin sections were treated with DNase-free Proteinase K at 37°C for 15 min, washed three times with PBS, and incubated with 3% H_2_O_2_ for 20 min. Sections were then labeled with biotin solution in the dark at 37°C for 60 min, followed by incubation with Streptavidin-HRP working solution at room temperature for 30 min. Finally, DAB substrate was applied for chromogenic detection, and results were visualized and photographed under a microscope.

**References**

[1] J. Chen, P. Gao, L. Peng, T. Liu, F. Wu, K. Xu, L. Chen, F. Tan, P. Xing, Z. Wang, J. Di, B. Jiang, X. Su. Downregulation of STK25 promotes autophagy via the Janus kinase 2/signal transducer and activator of transcription 3 pathway in colorectal cancer. *Mol Carcinog.* **2022**, 61, 572.

[2] L. Peng, M. Zhao, T. Liu, J. Chen, P. Gao, L. Chen, P. Xing, Z. Wang, J. Di, Q. Xu, H. Qu, B. Jiang, X. Su. A stop-gain mutation in GXYLT1 promotes metastasis of colorectal cancer via the MAPK pathway. *Cell Death Dis*. **2022**, 13, 395.

[3] T. Liu, M. Zhao, L. Peng, J. Chen, P. Xing, P. Gao, L. Chen, X. Qiao, Z. Wang, J. Di, H. Qu, B. Jiang, X. Su. WFDC3 inhibits tumor metastasis by promoting the ERβ-mediated transcriptional repression of TGFBR1 in colorectal cancer. *Cell Death Dis.* **2023**, 14, 425.

[4] X. Liu, C. Yan, C. Chang, F. Meng, W. Shen, S. Wang, Y. Zhang. FOXA2 Suppression by TRIM36 Exerts Anti-Tumor Role in Colorectal Cancer Via Inducing NRF2/GPX4-Regulated Ferroptosis. *Adv. Sci.* **2023**, 10, e2304521.
