# Supplementary material for: MTCH2 Deficiency Promotes E2F4/TFRC‐Mediated Ferroptosis and Sensitizes Colorectal Cancer Liver Metastasis to Sorafenib
Source: Adv Sci (Weinh). 2025 Jul 2;12(36):e00019. doi: 10.1002/advs.202500019 (PMC12463053; doi:10.1002/advs.202500019)
Supplement: Supplementary file 3 — Supporting Information [file ADVS-12-e00019-s001.docx]

**MTCH2 Deficiency Promotes E2F4/TFRC-Mediated Ferroptosis and Sensitizes Colorectal Cancer Liver Metastasis to Sorafenib**

*Pu Xing^1^, Jiangbo Chen^1^, Hao Hao^1^, Xiaowen Qiao^1^, Xinying Yang^1^, Kai Weng^1^, Jie Chen^2^,*

*Lin Song^1^, Tianqi Liu^1,3^, Yifan Hou^1^, Tongkun Song^1^, Yumeng Ran^1^, Bo Chen^1^, Hong Yang^1,4^, Wei Zhao^5^, Zaozao Wang^1^, Jiabo Di^1^, Beihai Jiang^1,*^, Xiangqian Su^1,6,*^*

*Corresponding authors.

**Supplementary Table S2.** Clinical information of 4 CRC samples for western blot.

| Sample ID | Gender | Age | Tumor location | T stage | N stage | M stage | Pathologic stage | Differentiation grade | Preoperative treatment |
| --- | --- | --- | --- | --- | --- | --- | --- | --- | --- |
| 1 | M | 62 | Hepatic Flexure | T4a | N1 | M1a | IVA | Middle | NO |
| 2 | F | 58 | Sigmoid Colon | T4b | N0 | M0 | IIB | Low | NO |
| 3 | F | 55 | Hepatic Flexure | T2 | N0 | M0 | I | Middle | NO |
| 4 | M | 64 | Sigmoid Colon | T4a | N0 | M0 | IIA | Middle | NO |
