# Supplementary material for: MTCH2 Deficiency Promotes E2F4/TFRC‐Mediated Ferroptosis and Sensitizes Colorectal Cancer Liver Metastasis to Sorafenib
Source: Adv Sci (Weinh). 2025 Jul 2;12(36):e00019. doi: 10.1002/advs.202500019 (PMC12463053; doi:10.1002/advs.202500019)
Supplement: Supplementary file 5 — Supporting Information [file ADVS-12-e00019-s004.docx]

**MTCH2 Deficiency Promotes E2F4/TFRC-Mediated Ferroptosis and Sensitizes Colorectal Cancer Liver Metastasis to Sorafenib**

*Pu Xing^1^, Jiangbo Chen^1^, Hao Hao^1^, Xiaowen Qiao^1^, Xinying Yang^1^, Kai Weng^1^, Jie Chen^2^,*

*Lin Song^1^, Tianqi Liu^1,3^, Yifan Hou^1^, Tongkun Song^1^, Yumeng Ran^1^, Bo Chen^1^, Hong Yang^1,4^, Wei Zhao^5^, Zaozao Wang^1^, Jiabo Di^1^, Beihai Jiang^1,*^, Xiangqian Su^1,6,*^*

*Corresponding authors.

**Supplementary Table S4.** Relevant primer sequences.

| Gene | Primer sequences | | Species |
| --- | --- | --- | --- |
| MTCH2 | Forward  Reverse | 5′- GGTCTTGTTCCTCGCCTTCT -3′  5′- TGGTAGAAACCCCACTGTCC -3′ | Human |
| E2F4 | Forward  Reverse | 5′- TGCCACCACCTGAAGATTT - 3′  5′- GGAGTGAGCTGAGGACTATTTG -3′ | Human |
| TFRC | Forward  Reverse | 5′- ATCGGTTGGTGCCACTGAATGG -3′  5′- ACAACAGTGGGCTGGCAGAAAC -3′ | Human |
| ACSL4 | Forward  Reverse | 5′- CATCCCTGGAGCAGATACTCT -3′  5′- TCACTTAGGATTTCCCTGGTCC -3′ | Human |
| SCD1 | Forward  Reverse | 5′- CCGGACACGGTCACCCGTTG -3′  5′- CGCCTTGCACGCTAGCTGGT -3′ | Human |
| DMT1 | Forward | 5′- CTAGACTGGGAGTGGTTACTGG -3′ | Human |
|  | Reverse | 5′- AGGATGACTCGTGGGACCTT -3′ |  |
| FTH1 | Forward | 5′- CCCCCATTTGTGTGACTTCAT -3′ | Human |
|  | Reverse | 5′- GCCCGAGGCTTAGCTTTCATT -3′ |  |
| SLC7A11 | Forward  Reverse | 5′- GGCTCCATGAACGGTGGTGTG -3′  5′- GCTGGTAGAGGAGTGTGCTTGC -3′ | Human |
| GPX4 | Forward  Reverse | 5′- CCGCTGTGGAAGTGGATGAAGATC -3′  5′- GCAGCCGTTCTTGTCGATGAGG -3′ | Human |
| ATF4 | Forward | 5′- CTCCGGGACAGATTGGATGTT -3′ | Human |
|  | Reverse | 5′- GGCTGCTTATTAGTCTCCTGGAC -3′ |  |
| YAP1 | Forward  Reverse | 5′- TAGCCCTGCGTAGCCAGTTA -3′  5′- TCATGCTTAGTCCACTGTCTGT -3′ | Human |
| IL-1β | Forward  Reverse | 5′- AGCTACGAATCTCCGACCAC -3′  5′- CGTTATCCCATGTGTCGAAGAA -3′ | Human |
| IL-6 | Forward  Reverse | 5′- CCTGAACCTTCCAAAGATGGC -3′  5′- TTCACCAGGCAAGTCTCCTCA -3′ | Human |
| GAPDH | Forward  Reverse | 5′-GGACTCATGACCACAGTCCATG -3′  5′- CAGGGATGATGTTCTGGAGAGC -3′ | Human |
| MTCH2 | Forward  Reverse | 5′- TCTTATGGCCGTCAACAACTG -3′  5′- GCACCAGCAATCTATCCAAGAA -3′ | Mouse |
| E2F4 | Forward | 5′- CATGAGTGGGCCTATCGAGG -3′ | Mouse |
|  | Reverse | 5′- GACTCTGGAGTAGATCGTCAGG -3′ |  |
| TFRC | Forward | 5′- GTTTCTGCCAGCCCCTTATTAT -3′ | Mouse |
|  | Reverse | 5′- GCAAGGAAAGGATATGCAGCA -3′ |  |
| GAPDH | Forward  Reverse | 5′- AGGTCGGTGTGAACGGATTTG -3′  5′- TGTAGACCATGTAGTTGAGGTCA -3′ | Mouse |
| E2F4-1-CHIP | Forward | 5′- CTGGCCTGCTGGTTACTGAC -3′ | Human |
|  | Reverse | 5′- TTGCCATCGCTGTTATTGGA -3′ |  |
| E2F4-2-CHIP | Forward | 5′- TGGAGGTTGGTCAAGGTCAG -3′ | Human |
|  | Reverse | 5′- CCAAGGGAAAAGGCGCCAAG -3′ |  |
| Loxp | Forward | 5′- GACTAAAGCTCTAAGGTGCCACAC -3′ | Mouse |
|  | Reverse | 5′-GAGGCATCAGGTCTAGATGAGACA -3′ |  |
| Cre | Forward | 5′- CCATAGGAAGCCAGTTTCCCTTC -3′ | Mouse |
|  | Reverse | 5′- TTCCAGGTATGCTCAGAAAACGC -3′ |  |
