# Supplementary material for: MTCH2 Deficiency Promotes E2F4/TFRC‐Mediated Ferroptosis and Sensitizes Colorectal Cancer Liver Metastasis to Sorafenib
Source: Adv Sci (Weinh). 2025 Jul 2;12(36):e00019. doi: 10.1002/advs.202500019 (PMC12463053; doi:10.1002/advs.202500019)
Supplement: Supplementary file 6 — Supporting Information [file ADVS-12-e00019-s008.docx]

**MTCH2 Deficiency Promotes E2F4/TFRC-Mediated Ferroptosis and Sensitizes Colorectal Cancer Liver Metastasis to Sorafenib**

*Pu Xing^1^, Jiangbo Chen^1^, Hao Hao^1^, Xiaowen Qiao^1^, Xinying Yang^1^, Kai Weng^1^, Jie Chen^2^,*

*Lin Song^1^, Tianqi Liu^1,3^, Yifan Hou^1^, Tongkun Song^1^, Yumeng Ran^1^, Bo Chen^1^, Hong Yang^1,4^, Wei Zhao^5^, Zaozao Wang^1^, Jiabo Di^1^, Beihai Jiang^1,*^, Xiangqian Su^1,6,*^*

*Corresponding authors.

**Supplementary Table S5.** Antibodies for western blot.

| Antibody | Manufacture | Item number | Species |
| --- | --- | --- | --- |
| MTCH2 | Proteintech, China | Cat#16888-1-AP | Rabbit |
| E2F4 | Abclonal, China | Cat#A19670 | Rabbit |
| TFRC | Abcam, UK | Cat#ab214039 | Rabbit |
| Ki67 | Cell Signaling Technology, USA | Cat#12202 | Rabbit |
| E-Cadherin | Cell Signaling Technology, USA | Cat#3195S | Rabbit |
| GPX4 | Abcam, UK | Cat#ab125066 | Rabbit |
| SLC7A11 | Abcam, UK | Cat#ab300667 | Rabbit |
| Flag | Sigma-Aldrich, USA | Cat# F1804 | Mouse |
| c-Myc | Clontech Laboratories, USA | Cat#631206 | Mouse |
| Ubiquitin | Santa Cruz Biotechnology, USA | Cat#sc-8017 | Mouse |
| β-actin | Sigma-Aldrich, USA | Cat#A1978 | Mouse |
| Histone H3 | Cell Signaling Technology, USA | Cat#4499S | Rabbit |
| TOM20 | Abclonal, China | Cat#A26784 | Rabbit |
| goat anti-mouse IgG | ZSGB-BIO, China | Cat#ZB-2305 | Goat |
| goat anti-rabbit IgG | ZSGB-BIO, China | Cat#ZB-2301 | Goat |
