# Supplementary material for: MTCH2 Deficiency Promotes E2F4/TFRC‐Mediated Ferroptosis and Sensitizes Colorectal Cancer Liver Metastasis to Sorafenib
Source: Adv Sci (Weinh). 2025 Jul 2;12(36):e00019. doi: 10.1002/advs.202500019 (PMC12463053; doi:10.1002/advs.202500019)
Supplement: Supplementary file 8 — Supporting Information [file ADVS-12-e00019-s009.pdf]

ADVANCED SCIENCE
Open Access

Supporting Information

for *Adv. Sci.*, DOI 10.1002/advs.202500019

MTCH2 Deficiency Promotes E2F4/TFRC-Mediated Ferroptosis and Sensitizes Colorectal Cancer Liver Metastasis to Sorafenib

Pu Xing, Jiangbo Chen, Hao Hao, Xiaowen Qiao, Xinying Yang, Kai Weng, Jie Chen, Lin Song, Tianqi Liu, Yifan Hou, Tongkun Song, Yumeng Ran, Bo Chen, Hong Yang, Wei Zhao, Zaozao Wang, Jiabo Di, Beihai Jiang and Xiangqian Su**

Supplementary Figure S1. Related to Figure 1.

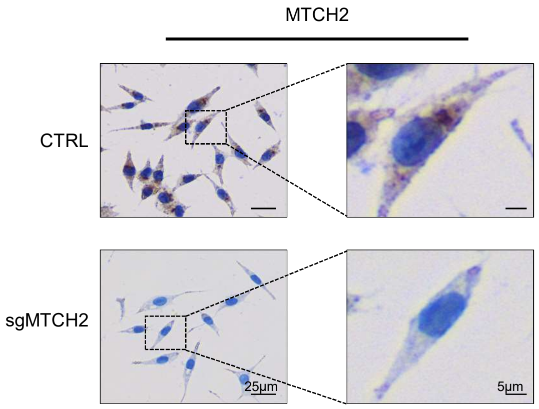

Supplementary Figure S1. Validation of the specificity of anti-MTCH2 antibody. Representative immunohistochemistry images showing MTCH2 expression in wild-type or MTCH2 knockout HCT116 cells. Scale bars, 25 μm and 5 μm.

Supplementary Figure S2. Related to Figure 2.

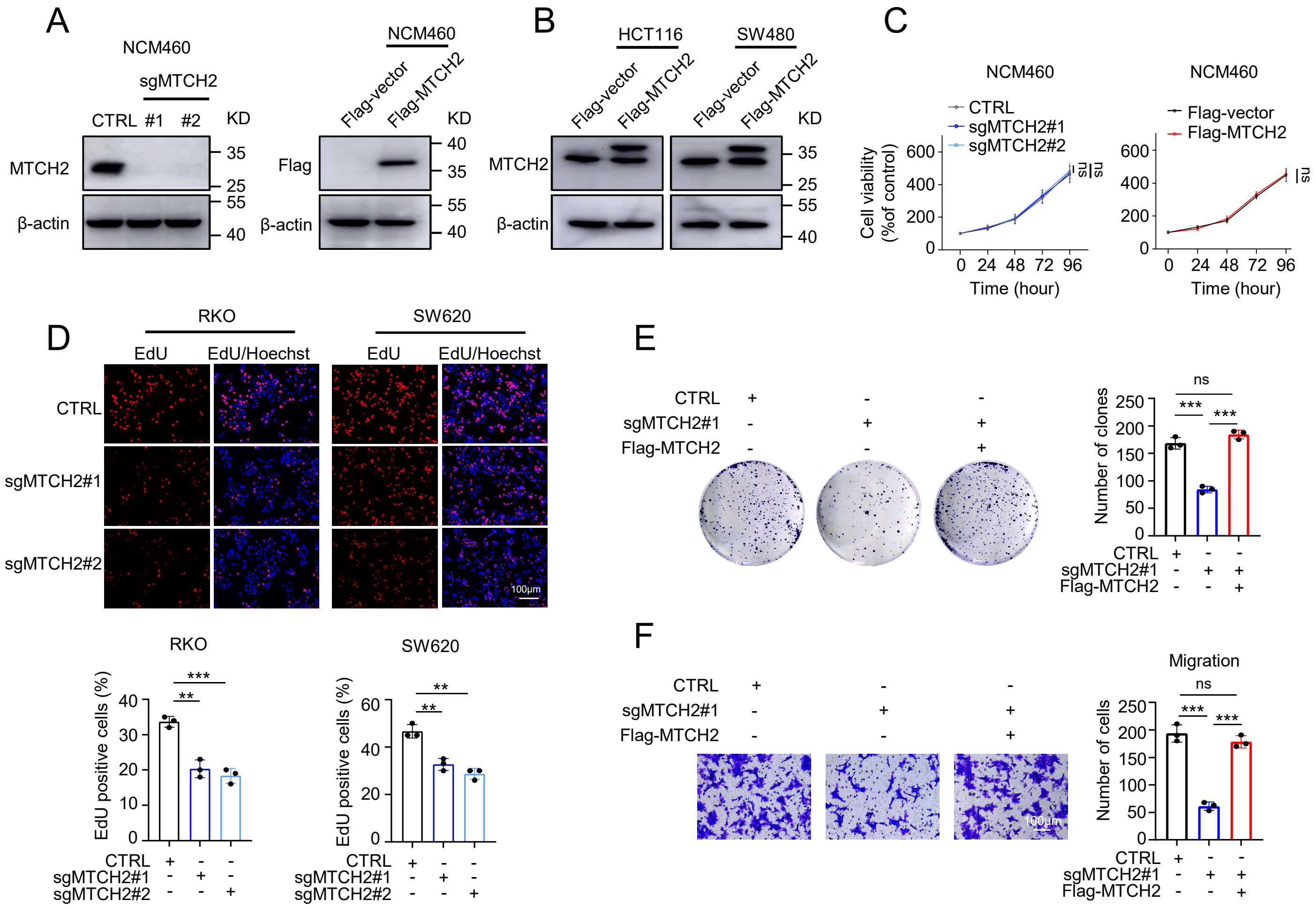

Supplementary Figure S2. MTCH2 knockout inhibits CRC cell proliferation. (A, B) Western blot assays showing the knockout and overexpression efficiency of MTCH2 in NCM460 cells (A) and CRC cells (B), respectively. **(C)** CCK-8 viability assays were performed after knockout and overexpression of MTCH2 in NCM460 cells. **(D)** Representative images were shown for 5-ethynyl-2'-deoxyuridine (EdU) and Hoechst staining in MTCH2 knockout CRC cells, and quantifications of EdU-positive tumor cells were presented in a bar graph. Scale bar, 100μm. **(E, F)** Clone formation (E) and Transwell (F) assays of HCT116 cells transfected with sgMTCH2#1 and Flag-MTCH2 rescue. Scale bar, 100μm. Statistics were performed using unpaired Student's t-test. Data are presented as the means ± SD of at least three independent experiments. $^{**}P < 0.01$, $^{***}P < 0.001$; ns, not significant.

Supplementary Figure S3. Related to Figure 3.

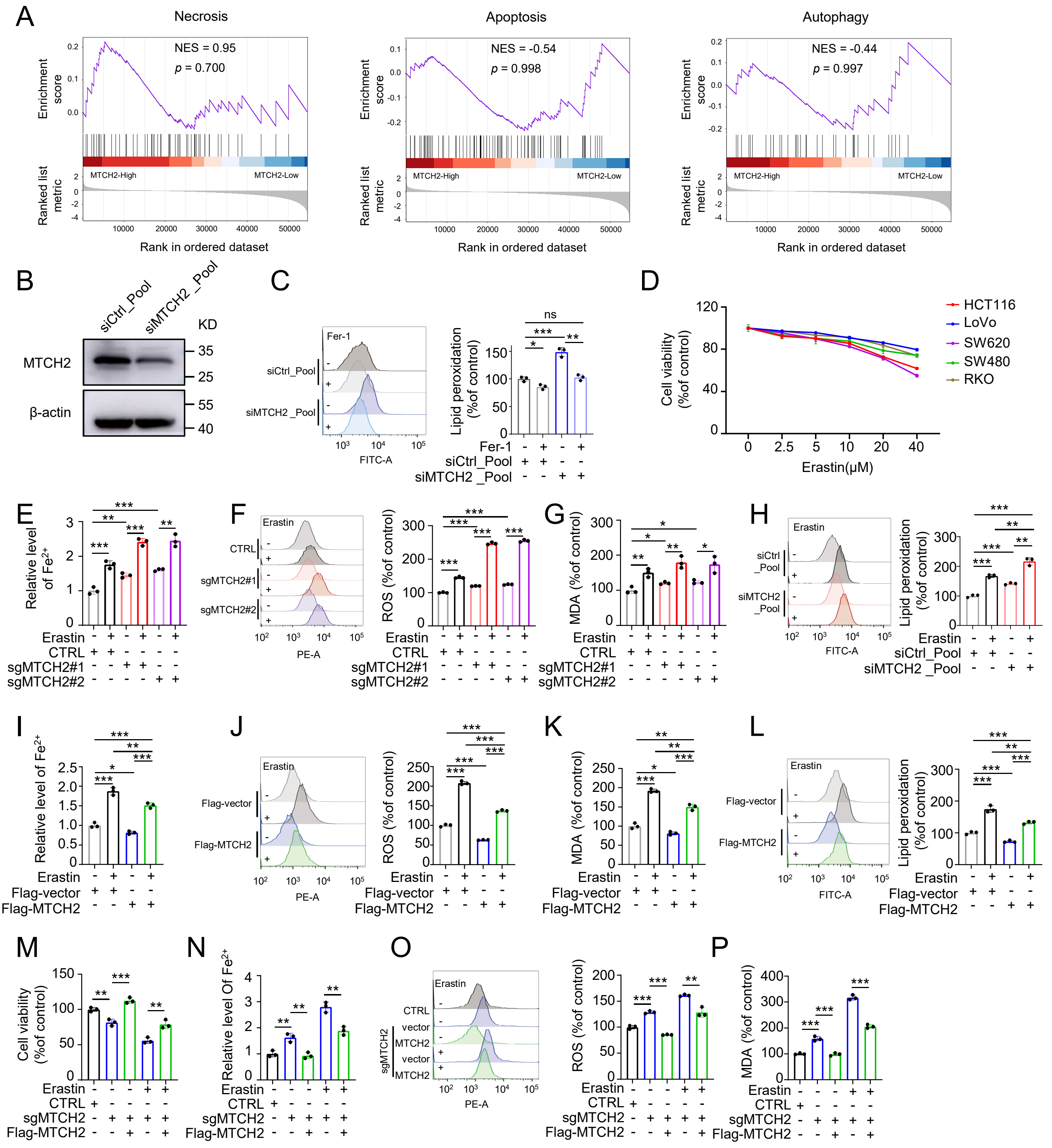

Supplementary Figure S3. MTCH2 overexpression inhibits erastin-induced ferroptosis in CRC cells. (A) GSEA of necrosis, apoptosis, and autophagy based on differentially expressed genes related to MTCH2 expression in CRC data from TCGA. **(B)** Verification of siMTCH2_Pool knockdown efficiency in HCT116 cells. **(C)** Lipid peroxidation levels were assessed in HCT116 cells via the BODIPY 581/591 C11 probe. **(D)** CCK-8 viability assays of CRC cells exposed to the indicated concentrations of erastin for 48 hours. **(E-H)** Levels of Fe^{2+} (E), ROS (F), MDA (G), and lipid peroxidation (H) in sgRNA control or sgMTCH2 CRC cells treated with erastin (20 μM). **(I-L)** Levels of Fe^{2+} (I), ROS (J), MDA (K), and lipid peroxidation (L) in MTCH2-overexpressing SW480 cells treated with erastin (20 μM). **(M-P)** Cell viability (M) and levels of Fe^{2+} (N), ROS (O), and MDA (P) of HCT116 cells followed by indicated treatment. Two-way ANOVA with Tukey's multiple comparisons test was used for statistical analysis. The results shown indicate the means ± SD of at least three independent replicates. **P* < 0.05, ***P* < 0.01, ****P* < 0.001; ns, not significant.

Supplementary Figure S4. Related to Figure 4.

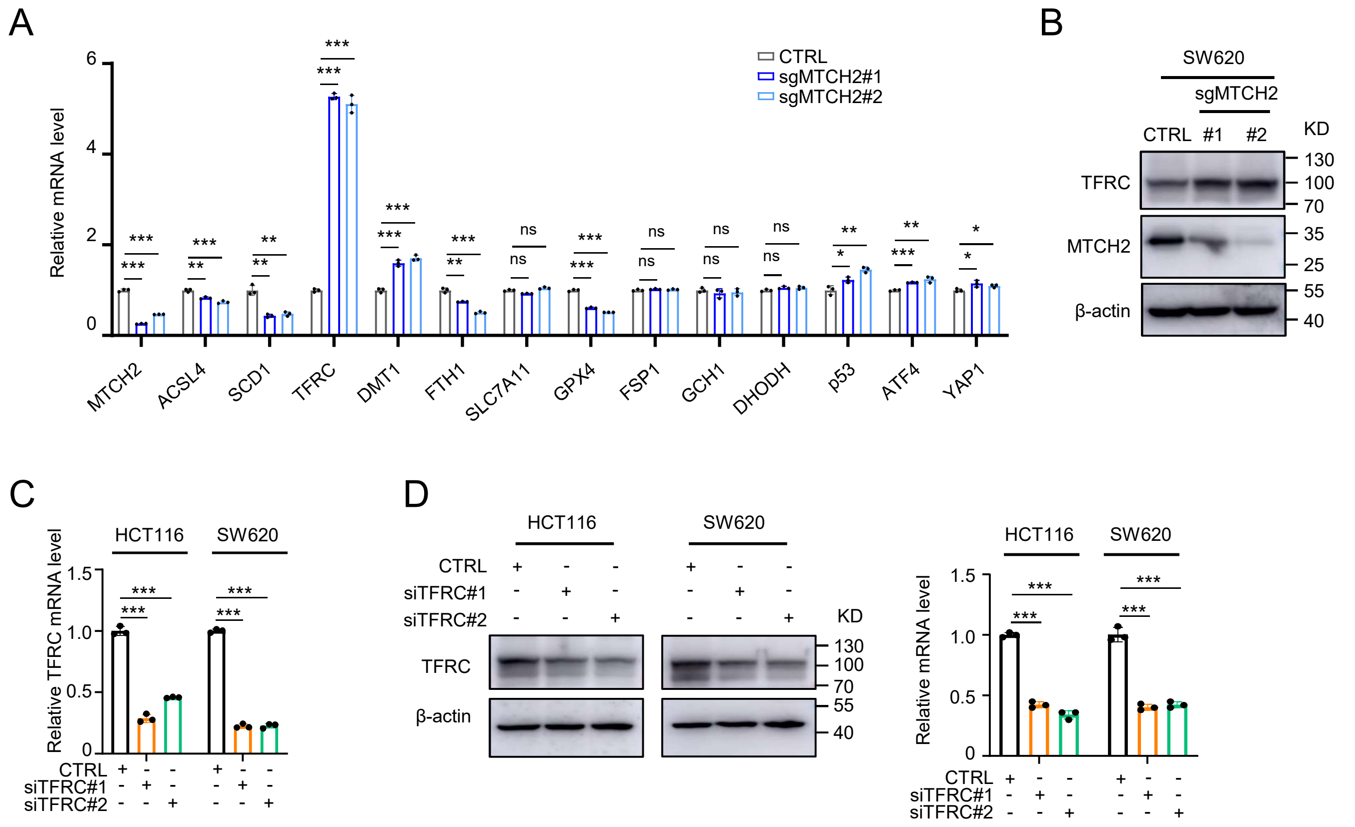

Supplementary Figure S4. Identification of ferroptosis-associated factors in MTCH2-depleted CRC cells. (A) qRT-PCR analysis of relative mRNA expression of 13 ferroptosis-associated genes in MTCH2-deficient HCT116 cells. **(B)** The protein expression levels of TFRC and MTCH2 were detected in MTCH2-depleted SW620 cells using Western blot. **(C, D)** qRT-PCR (C) and Western blot (D) analyses to validate the knockdown efficiency of TFRC-siRNA in HT116 and SW620 cells. Statistics were performed using unpaired Student's t-test. Data are presented as the means ± SD of at least three independent experiments. *$P < 0.05$, **$P < 0.01$, ***$P < 0.001$; ns, not significant.

Supplementary Figure S5. Related to Figure 5.

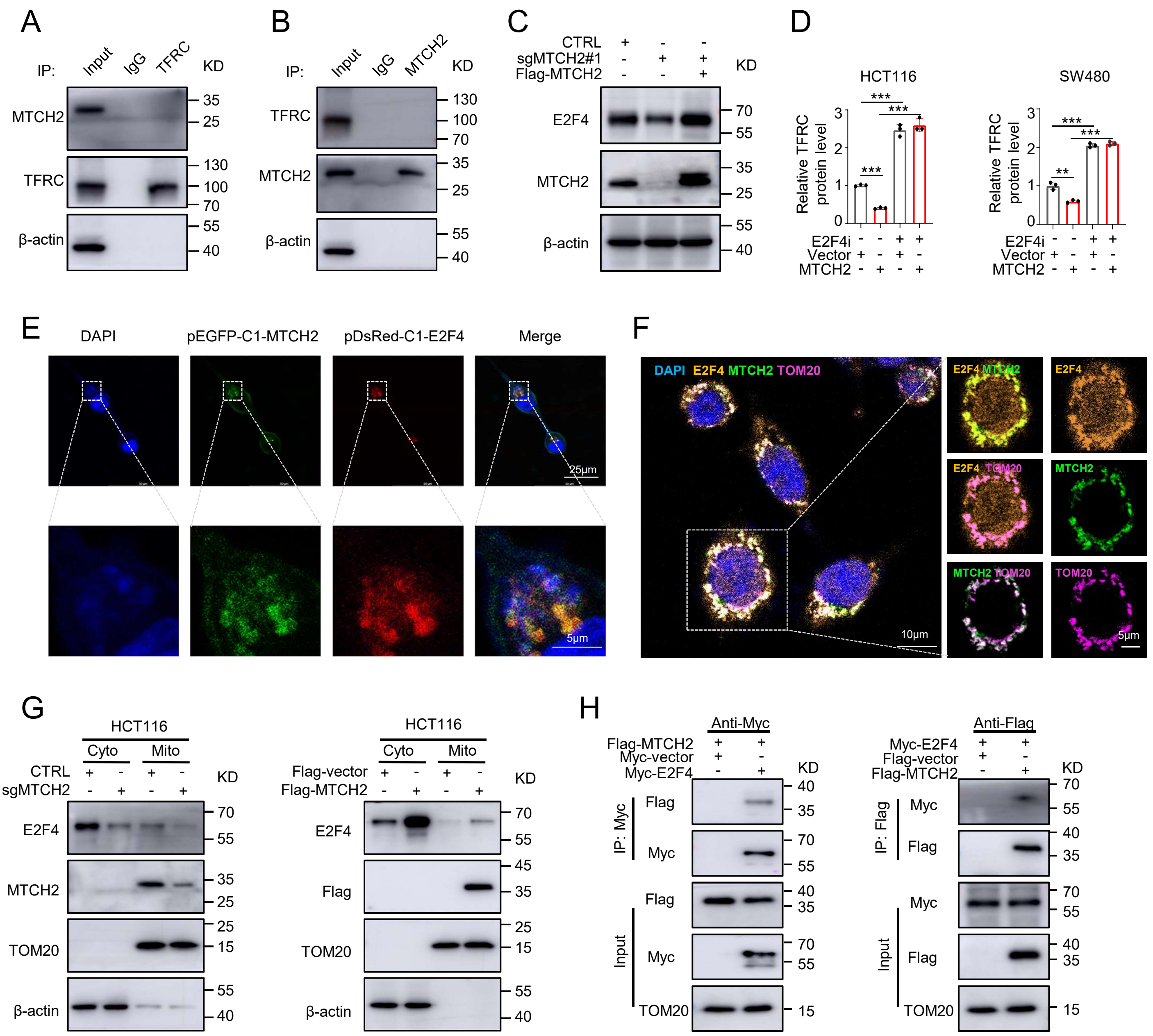

Supplementary Figure S5. MTCH2 interacts with E2F4 in mitochondria. (A) Immunoprecipitation of the MTCH2 protein by an anti-TFRC antibody in RKO cells. **(B)** Immunoprecipitation of the TFRC protein by an anti-MTCH2 antibody in RKO cells. **(C)** Western blot analysis of E2F4 and MTCH2 levels of HCT116 cells transfected with sgMTCH2#1 and Flag-MTCH2 rescue. **(D)** Quantification of relative protein levels normalized to β-actin in Figure 5H. Bar graphs indicate the relative protein levels compared with β-actin. **(E)** HCT116 cells co-transfected with pEGFP-C1-MTCH2 (green) and pDsRed-C1-E2F4 (red) were observed under a confocal microscope. Colocalization was shown by the merge (yellow). Scale bars, 25 μm and 5 μm. **(F)** Confocal images showing the co-localization of E2F4 (orange) with both MTCH2 (green) and TOM20 (purple) in HCT116 cells. Scale bars, 10 μm and 5 μm. **(G)** Distributions of E2F4 and MTCH2 in cytoplasmic and mitochondrial fractions of sgMTCH2 (left panels) and MTCH2-overexpressing (right panels) HCT116 cells. β-actin and TOM20 were used as cytoplasmic and mitochondrial markers, respectively. **(H)** Western blot of co-IP assays of exogenous MTCH2 and E2F4 using anti-Myc antibody to pull down Myc-E2F4 (left) or anti-Flag antibody to pull down Flag-MTCH2 (right) from mitochondrial lysates of HCT116 cells transfected as indicated. Blots were probed using the indicated antibodies. The statistical analysis was calculated by two-way ANOVA for multiple comparisons. Data present means ± SD from three independent experiments. $^{**}P < 0.01$, $^{***}P < 0.001$.

Supplementary Figure S6. Related to Figure 7.

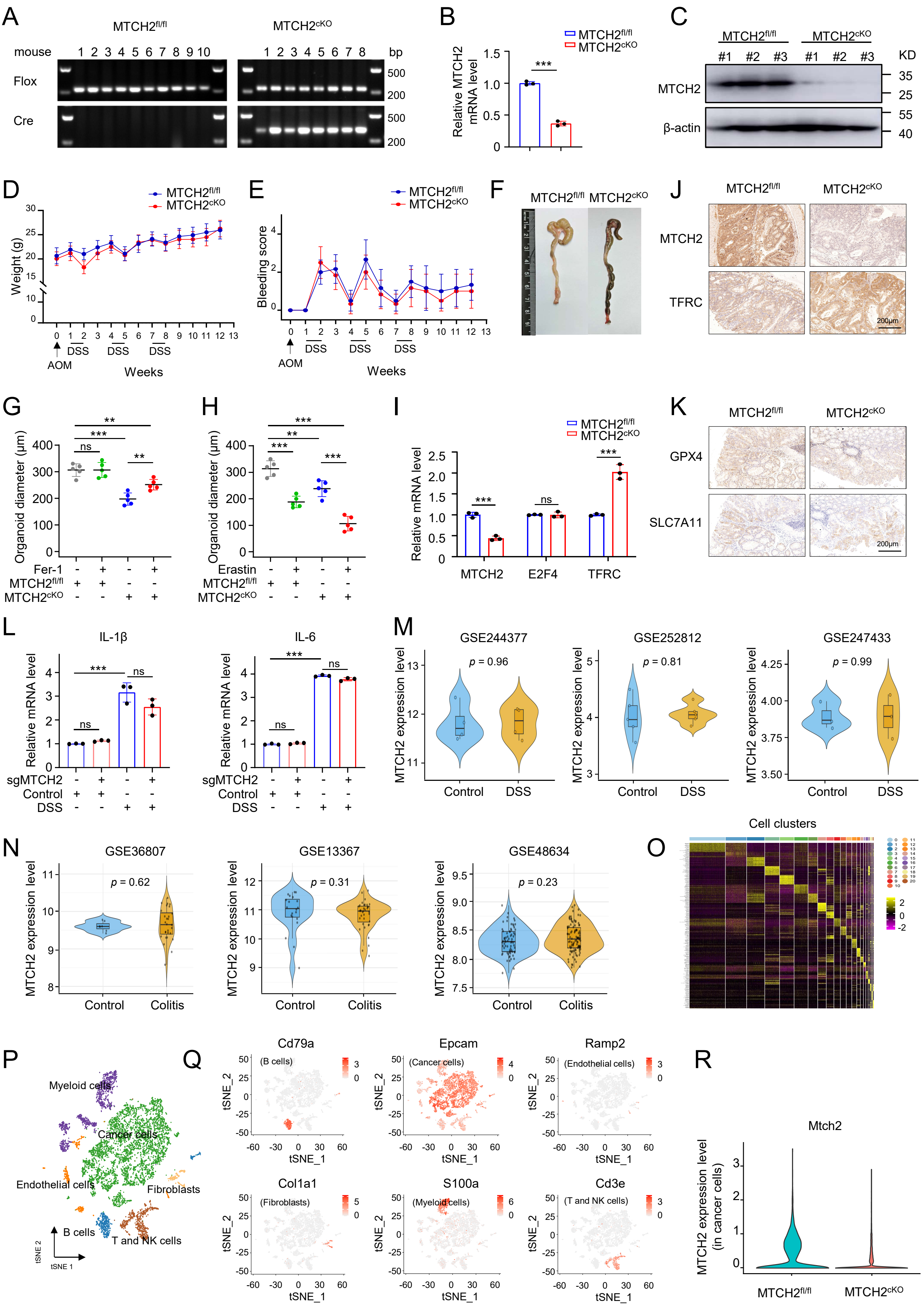

Supplementary Figure S6. Related to Figure 7.

Supplementary Figure S6. Conditional knockout of MTCH2 inhibits AOM/DSS-induced CRC in $MTCH2^{cKO}$ mice. (A) Genotyping based on agarose gel electrophoresis of PCR products amplified from mouse tail DNA. **(B, C)** Verification of MTCH2 knockout efficiency in colon tissues of $MTCH2^{fl/fl}$ and $MTCH2^{cKO}$ mice using qRT-PCR (B) and Western blot (C). **(D, E)** Body weight (D) and bleeding scores (E) in $MTCH2^{fl/fl}$ and $MTCH2^{cKO}$ mice during AOM/DSS treatment. **(F)** Representative images of colons from $MTCH2^{fl/fl}$ and $MTCH2^{cKO}$ mice (n = 5 per group). **(G, H)** Quantification of organoid diameter in Figure 7L (G) and Figure 7M (H). n = 5 for each group. **(I)** qRT-PCR analysis of relative mRNA **e**xpression levels of MTCH2, E2F4, and TFRC in colorectal tumors from the two groups. **(J, K)** Representative IHC staining images of MTCH2 and TFRC (J), as well as GPX4 and SLC7A11 (K) in CRC tumors from the two groups. Scale bar, 200 μm. **(L)** The levels of IL-1β and IL-6 were measured using qRT-PCR after NCM460 cells treated with 2% DSS for 24 h. **(M, N)** Analysis of MTCH2 expression in the DSS-induced colitis mouse models (M) and human colitis tissues (N) from the GEO databases. **(O)** Unsupervised clustering of 21 cell groups based on gene expression patterns. **(P)** tSNE visualization of six distinct cell clusters of CRC tumor tissues based on gene expression patterns. **(Q)** Feature plots showing conventional marker genes for different cell types, including Cd79a, Epcam, Ramp2, Col1a1, S100a9, and Cd3e. **(R)** The violin plots showing MTCH2 expression in tumor cells of $MTCH2^{fl/fl}$ and $MTCH2^{cKO}$ mice. The statistical analysis was calculated by Two-tailed Student's t-test and one-way ANOVA with Tukey's honest difference post hoc test. Data present means ± SD from three independent experiments. $^{**}P < 0.01$, $^{***}P < 0.001$; ns, not significant.

Supplementary Figure S7. Related to Figure 8.

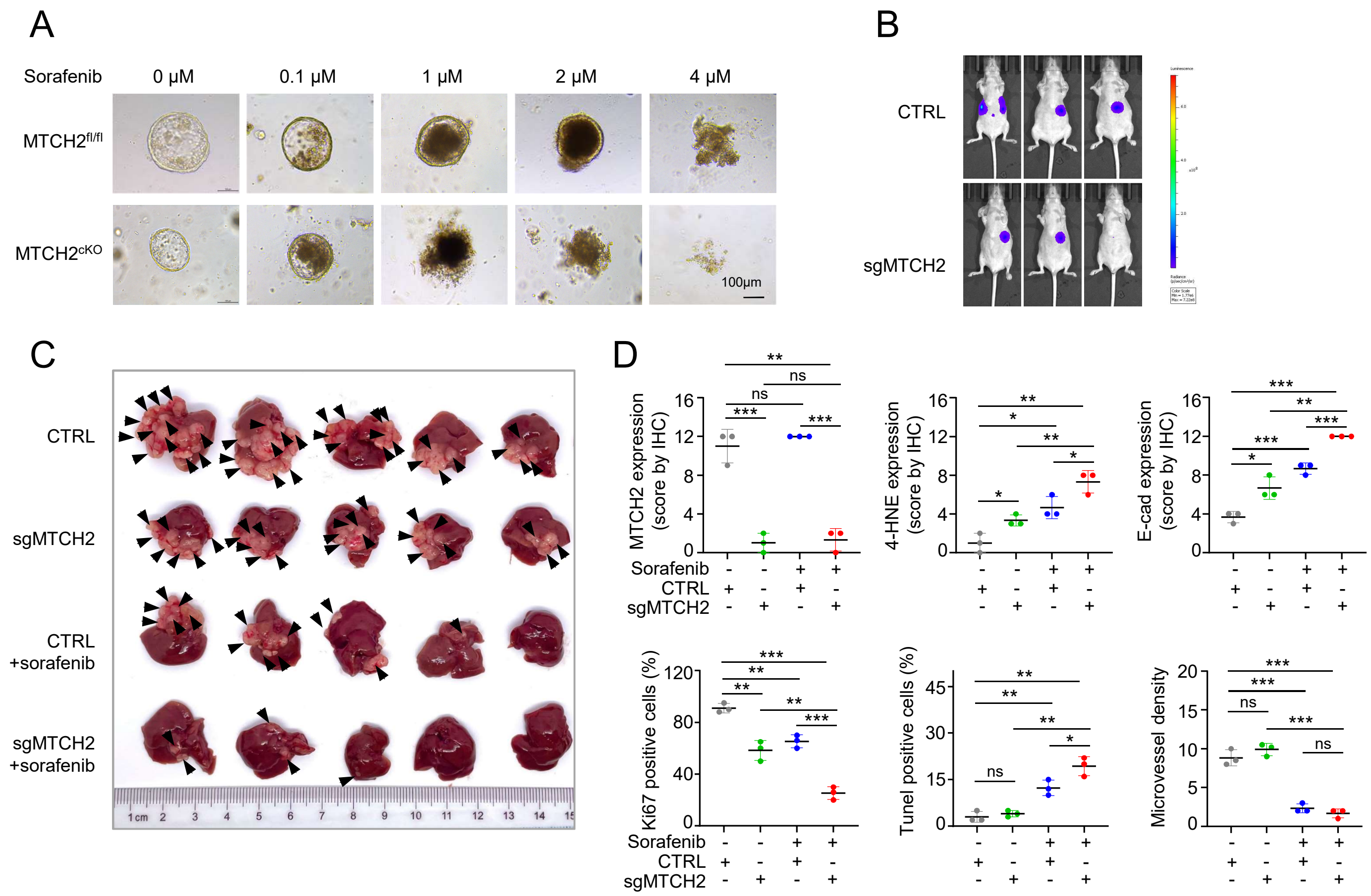

Supplementary Figure S7. Depletion of MTCH2 combined with sorafenib has synergistic effects on liver metastasis. (A) Representative image of MTCH2$^{fl/fl}$ and MTCH2^{cKO} mouse tumor organoids exposed to the indicated concentrations of sorafenib for 72 hours. Scale bar, 100 μm. **(B)** Representative bioluminescence imaging of mice from each treatment group 2 weeks after implantation of HCT116 cells. **(C)** Photographs of metastatic foci in the livers of mice from the indicated groups (n = 5 per group). The liver metastatic lesions are indicated by the black arrow. **(D)** Quantification of immunohistochemical analysis in Figure 8P (n = 3 for each group). The statistical analysis was calculated by two-way ANOVA for multiple comparisons. Data present means ± SD from three independent experiments. **P* < 0.05, ***P* < 0.01, ****P* < 0.001; ns, not significant.
