# Supplementary material for: MTCH2 Deficiency Promotes E2F4/TFRC‐Mediated Ferroptosis and Sensitizes Colorectal Cancer Liver Metastasis to Sorafenib
Source: Adv Sci (Weinh). 2025 Jul 2;12(36):e00019. doi: 10.1002/advs.202500019 (PMC12463053; doi:10.1002/advs.202500019)

ADVANCED SCIENCE
Open Access

Supporting Information

for *Adv. Sci.*, DOI 10.1002/advs.202500019

MTCH2 Deficiency Promotes E2F4/TFRC-Mediated Ferroptosis and Sensitizes Colorectal Cancer Liver Metastasis to Sorafenib

Pu Xing, Jiangbo Chen, Hao Hao, Xiaowen Qiao, Xinying Yang, Kai Weng, Jie Chen, Lin Song, Tianqi Liu, Yifan Hou, Tongkun Song, Yumeng Ran, Bo Chen, Hong Yang, Wei Zhao, Zaozao Wang, Jiabo Di, Beihai Jiang and Xiangqian Su**

Full unedited blot for Figure 1

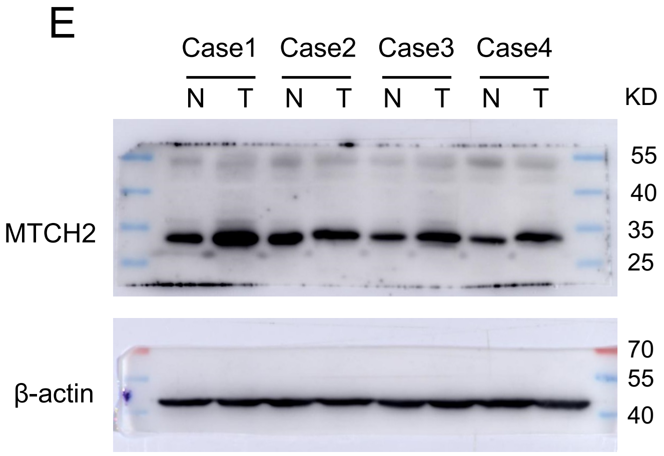

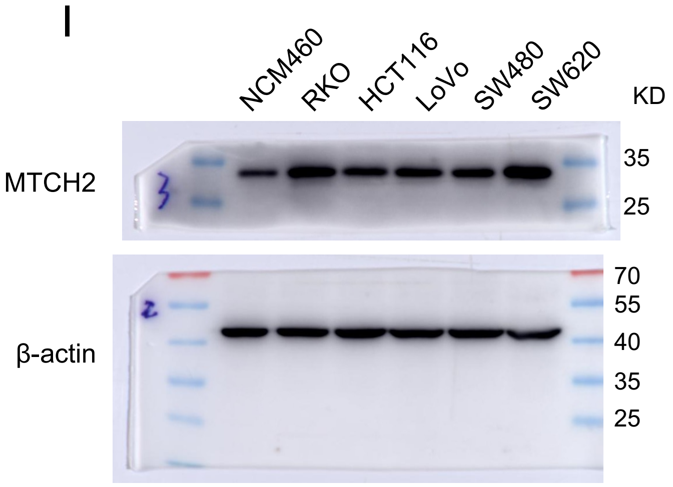

Full unedited blot for Figure 2

A

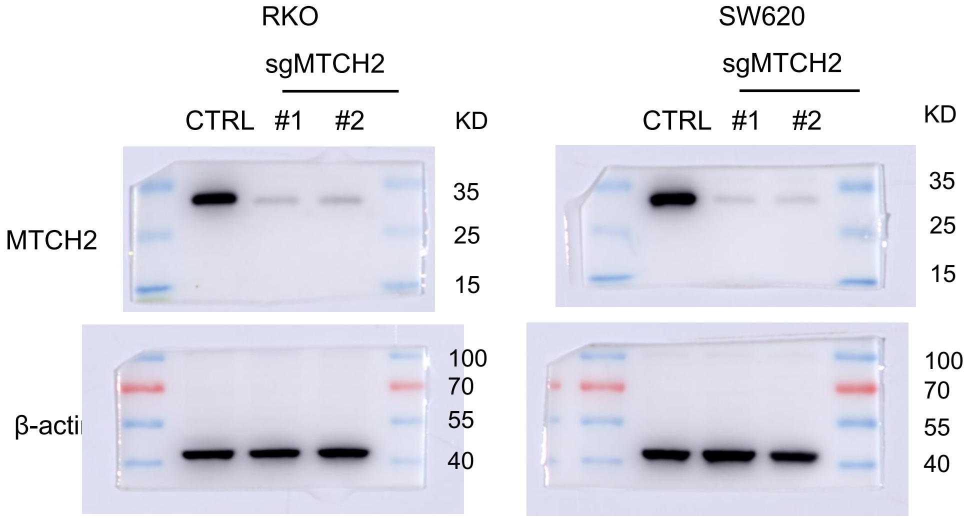

H

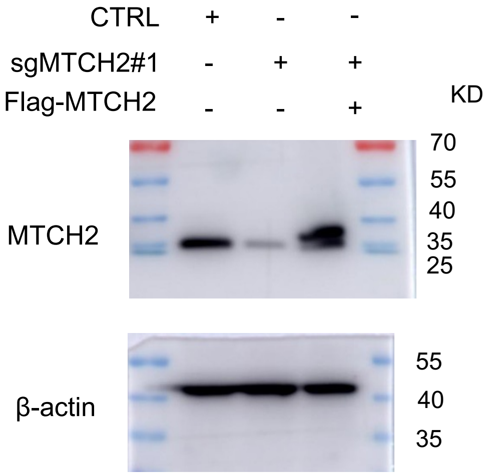

Full unedited blot for Figure 4

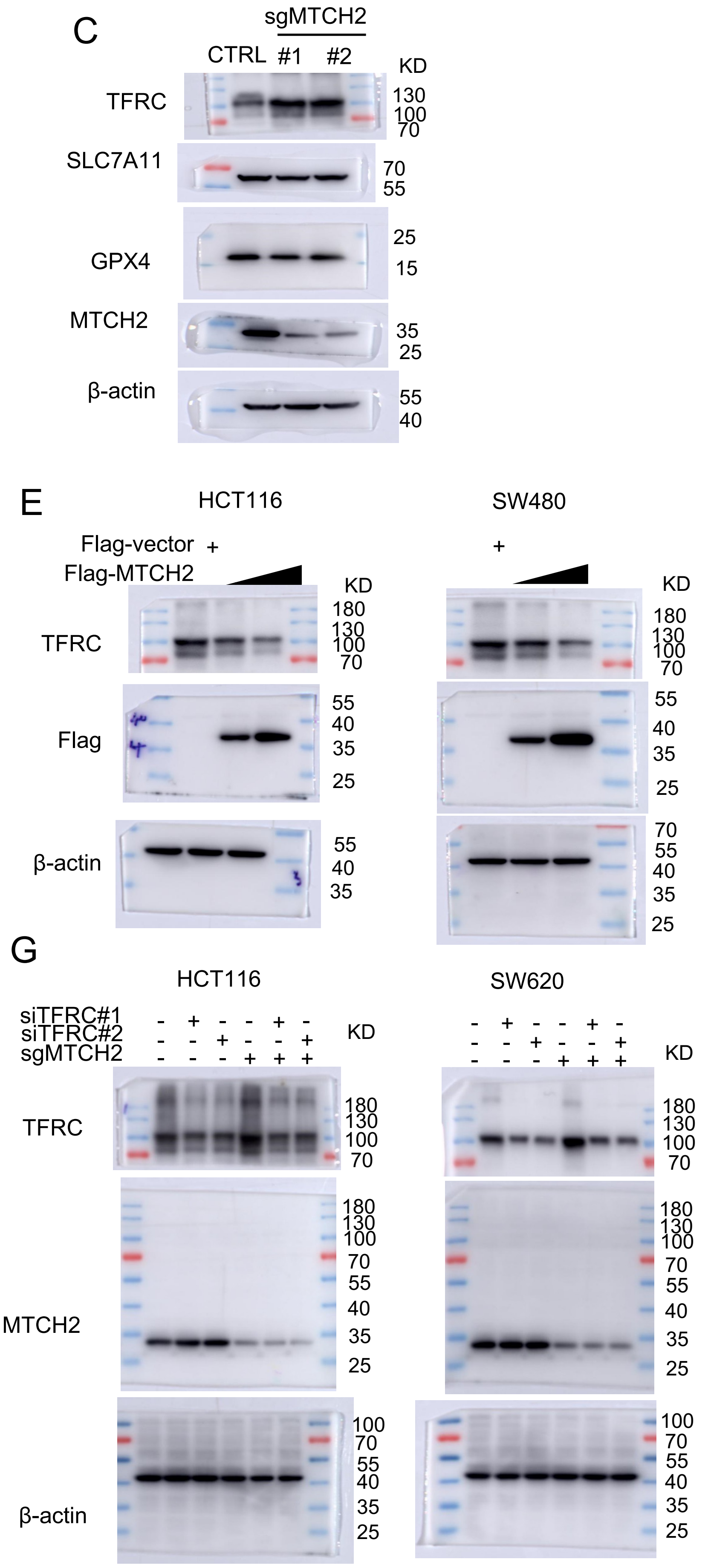

Full unedited blot for Figure 5

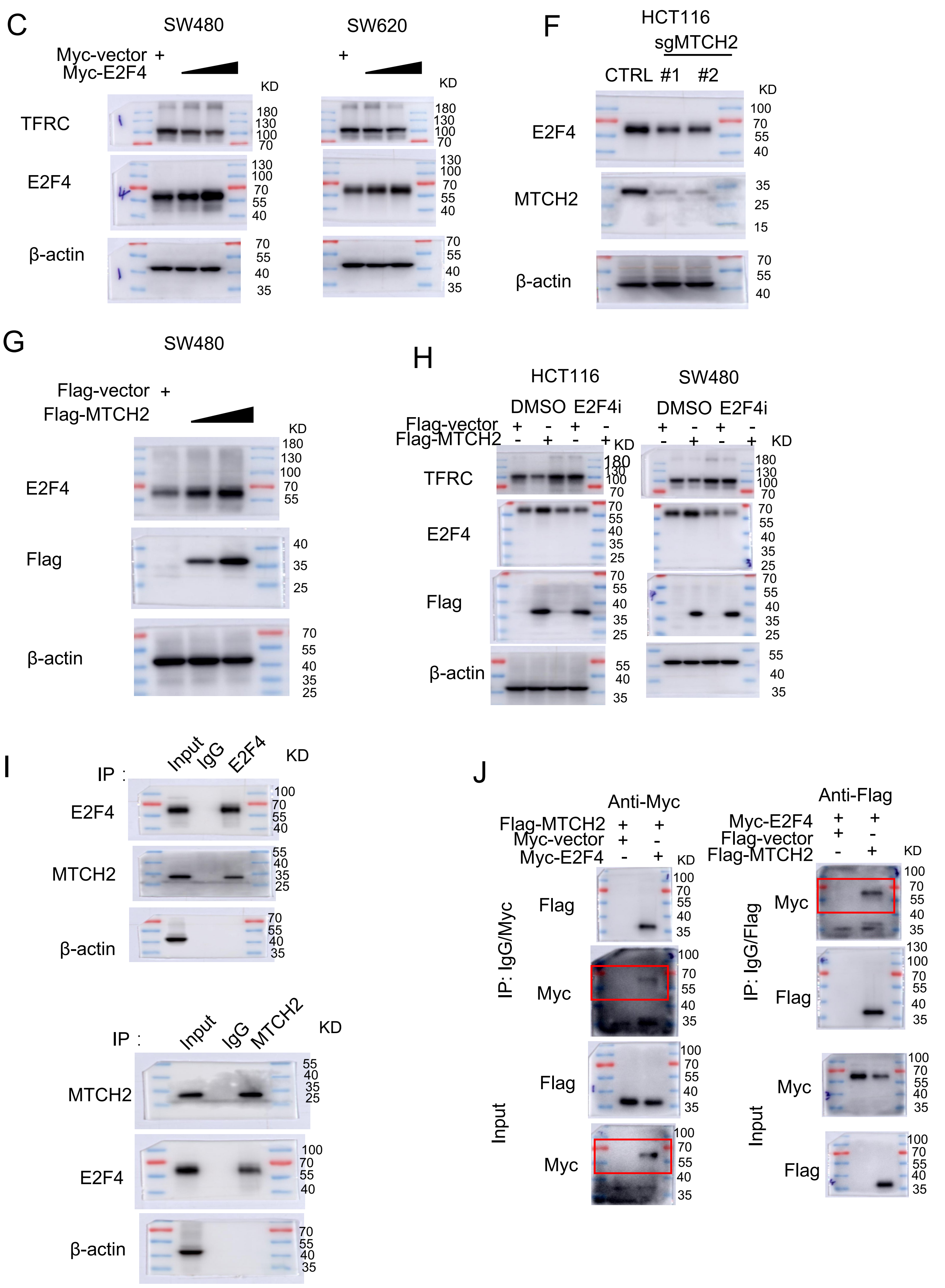

Full unedited blot for Figure 5 (continued)

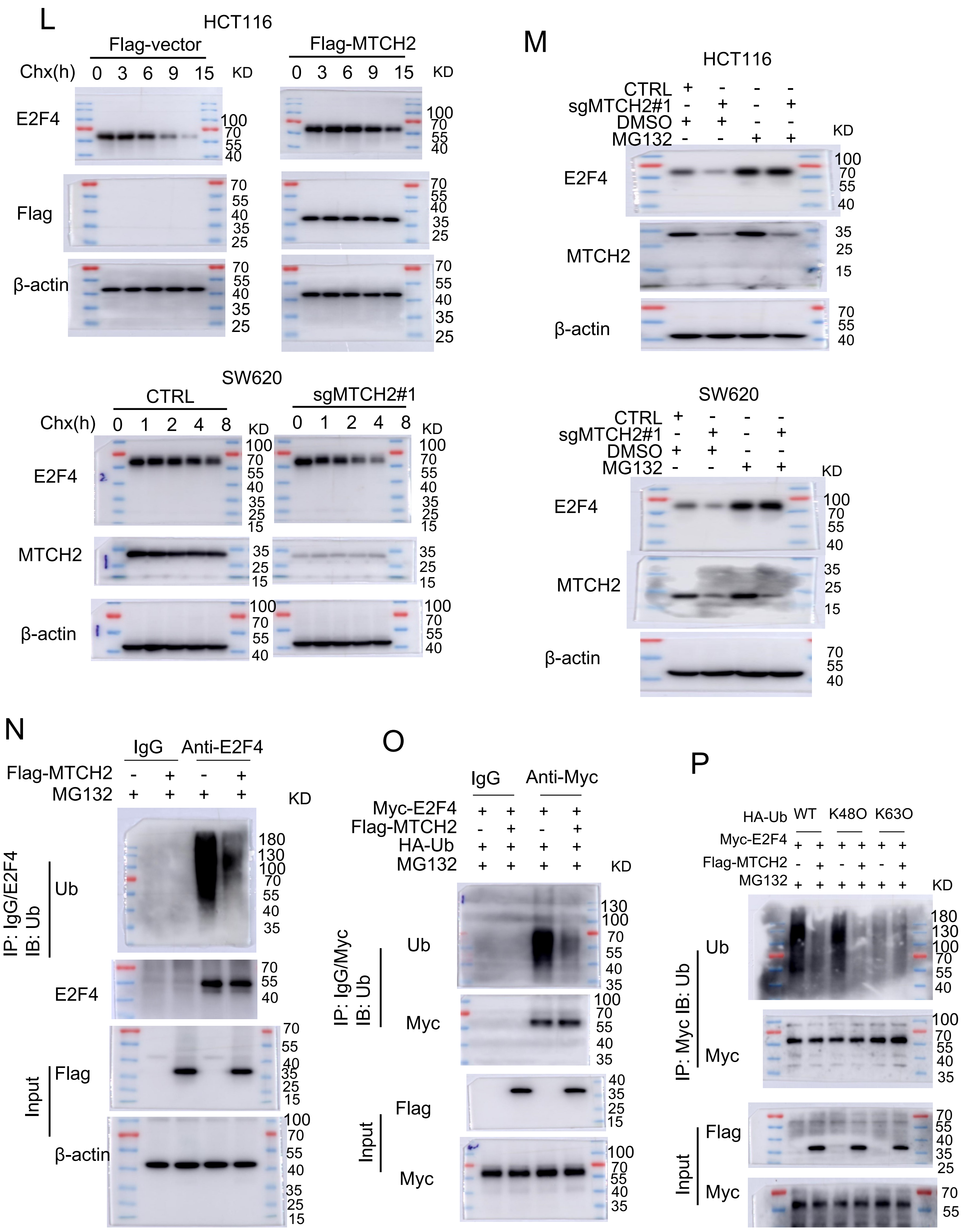

Full unedited gel for Figure 6

A

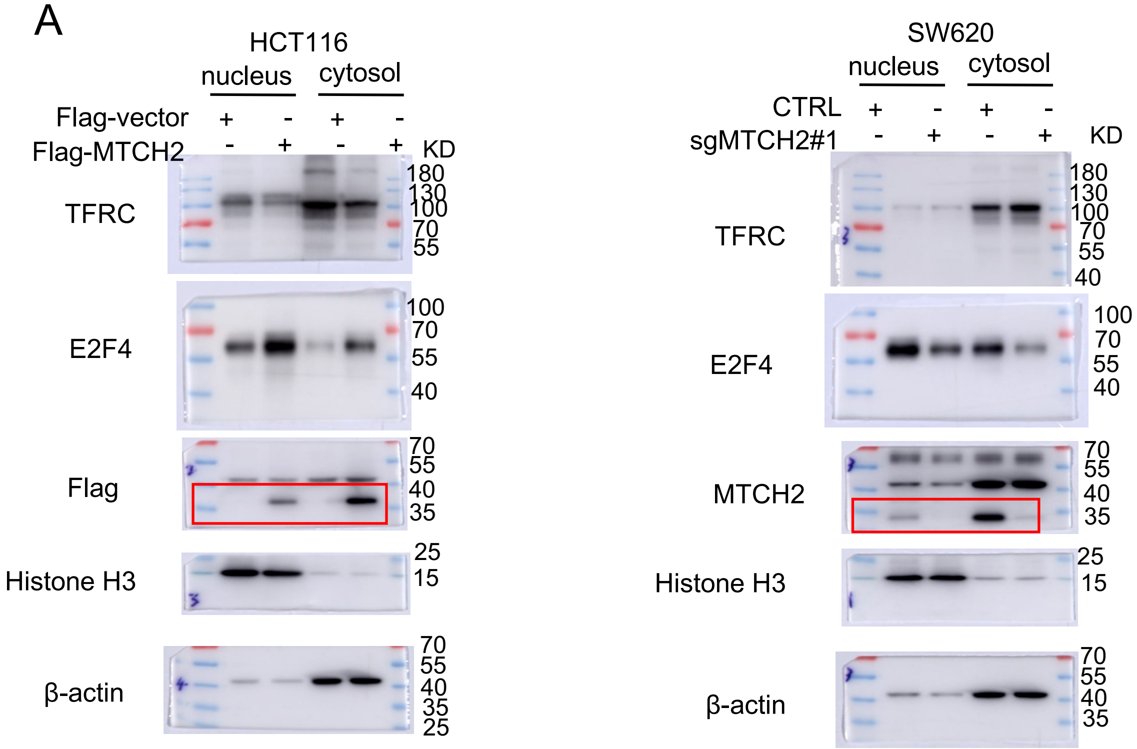

C

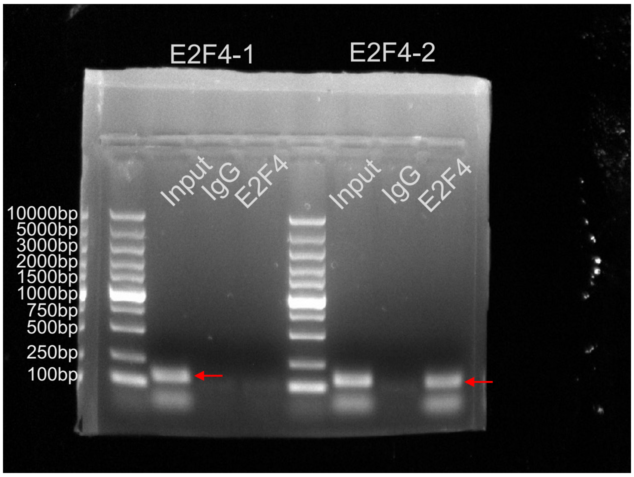

Q

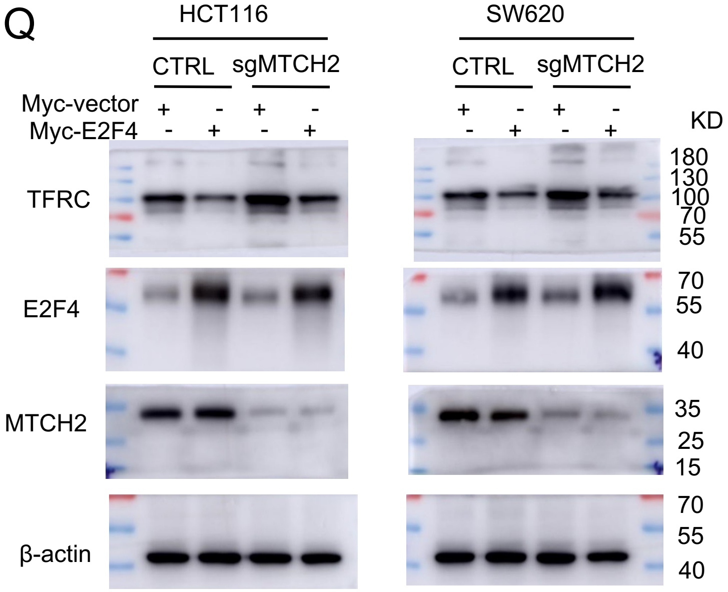

Full unedited blot for Figure 7

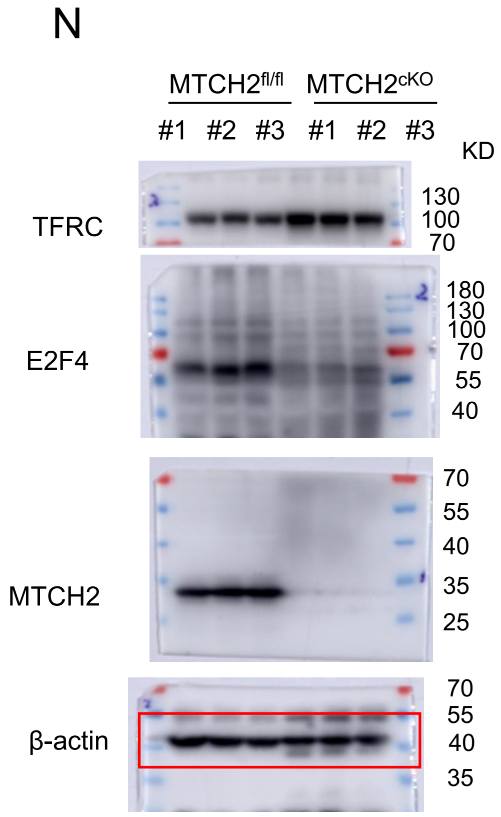

Full unedited blot for Supplementary Figure S2

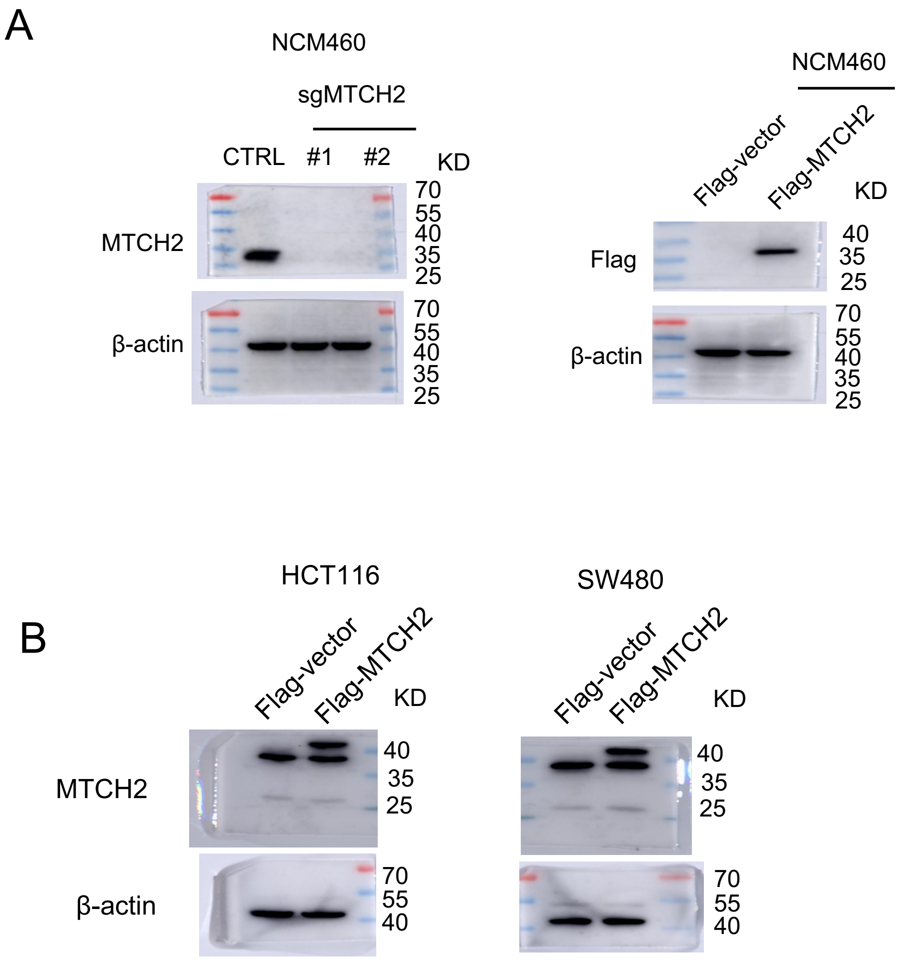

Full unedited blot for Supplementary Figure S3

B

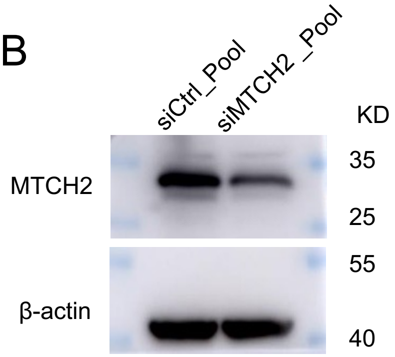

Full unedited blot for Supplementary Figure S4

B

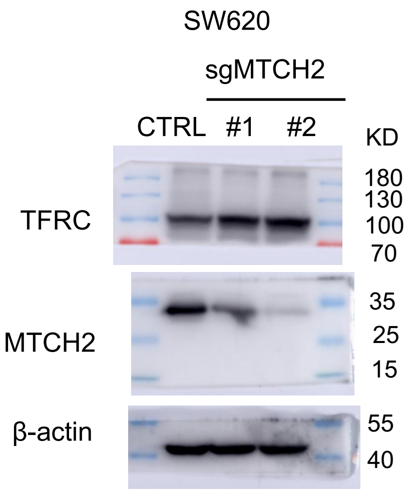

D

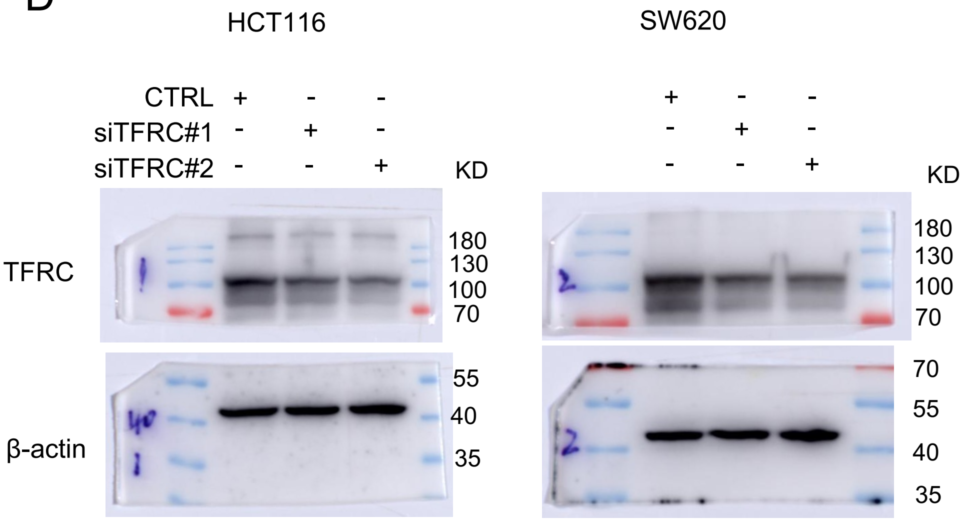

Full unedited blot for Supplementary Figure S5

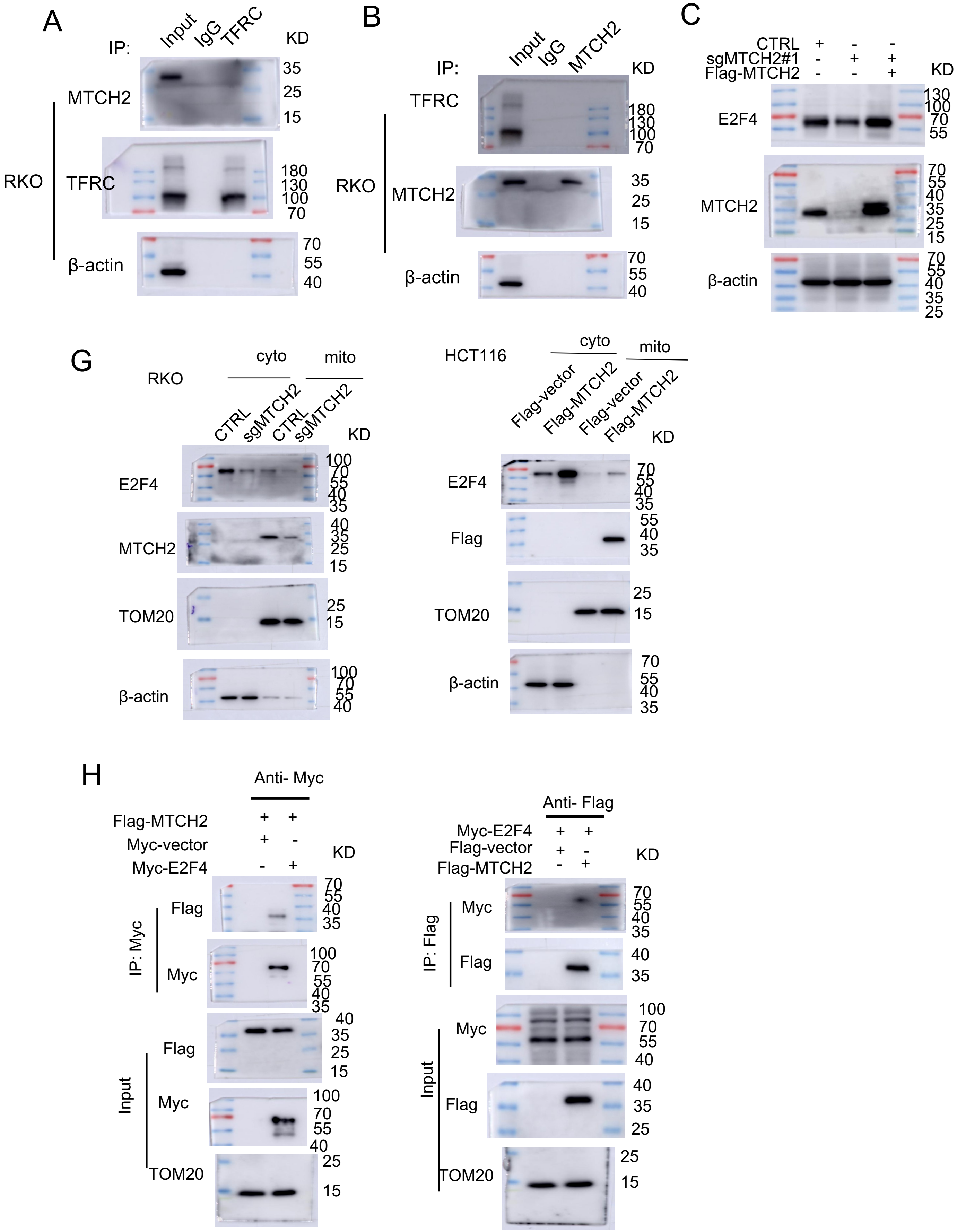

Full unedited blot/gel for Supplementary Figure S7

A

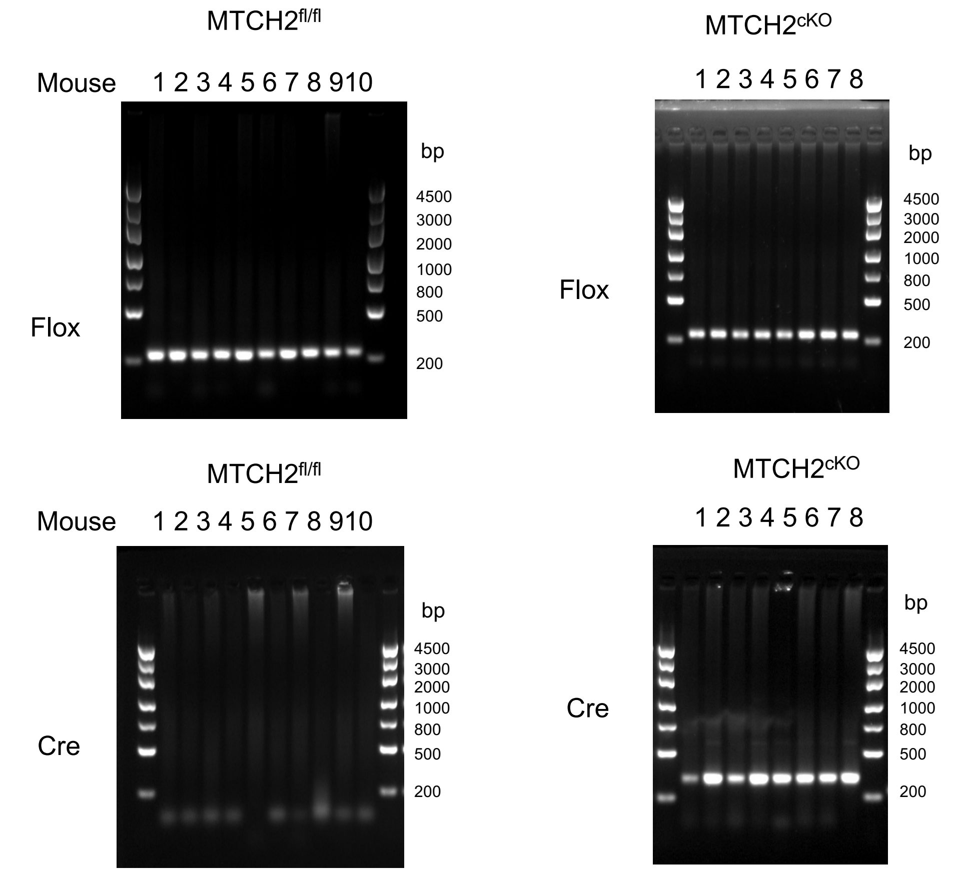

C

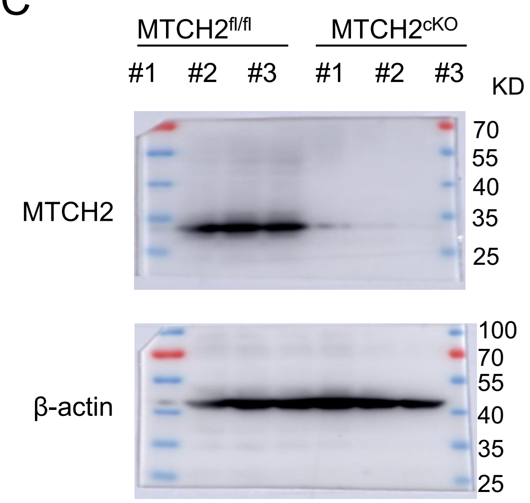

Supplement: Supplementary file 9 — Supporting Information [file ADVS-12-e00019-s007.pdf]
